# Endocrine and aggressive responses to competition are moderated by contest outcome, gender, individual versus team competition, and implicit motives

**DOI:** 10.1371/journal.pone.0181610

**Published:** 2017-07-27

**Authors:** Jon K. Oxford, Johanna M. Tiedtke, Anna Ossmann, Dominik Özbe, Oliver C. Schultheiss

**Affiliations:** 1 Behavioral & Social Sciences, Southern Arkansas University, Magnolia, Arkansas, United States of America; 2 Department of Psychology, Friedrich-Alexander University, Erlangen, Germany; Waseda University, JAPAN

## Abstract

This study examined hormonal responses to competition in relation to gender, social context, and implicit motives. Participants (*N* = 326) were randomly assigned to win or lose in a 10-round, virtual face-to-face competition, in same-sex individual- and team-competition contexts. Saliva samples, taken before and twice after the competition, were assayed for testosterone (T), estradiol (E), progesterone (P), and cortisol (C). Implicit needs for power (nPower) and affiliation (nAffiliation) were assessed with a picture-story exercise before the competition. Aggression was measured via the volume at which participants set noise blasts for their opponents. Men competing individually and women competing as teams showed similar T increases after winning. C was differentially associated with outcome in the team matches, with higher post-match cortisol for winning women, and an opposite effect for male teams. Analyses including implicit motives indicated that situational variables interacted with motivational needs in shaping hormonal responses to competition: in naturally cycling women, nPower predicted T increases after winning and T and E decreases after losing. In men, nPower predicted T increases after losing and decreases after winning. In male teams, nPower predicted C increases after losing, but not after winning, whereas in individual competitions, nPower was a general negative predictor of C changes in women. nAffiliation predicted P increases for women competing as teams, and P decreases for women competing individually. Aggression was higher in men, losers, and teams than in women, winners, and individuals. High aggression was associated with high baseline C in women competing individually and with low baseline C and C decreases in women competing as teams and in men generally. Our findings suggest that while situational and gender factors play a role in hormonal responses to competition, they also depend on their interplay with motivational factors. They also suggest that while aggression is strongly affected by situational factors in the context of a competition, it has no direct association with motivational and hormonal correlates of dominance (nPower, T, E) and instead is associated with (mostly) low levels of C.

## Introduction

Research on hormones and dominance in humans relies heavily on animal models of dominance attained or lost during competitive interactions, focusing primarily on T and predicting T increases after winning and T decreases after losing a competition [1], [2]. However, this research is plagued by several problems. One is the idea that the outcome of social situations (victory or defeat) is the only or primary factor in hormone changes. Contrary to this notion, however, many well-designed studies on human subjects failed to find such a direct effect of the social situation on hormones [3] and instead suggest that attributes of the person, such as her or his motivational need for power, moderate hormonal responses to competition [4]. Another potential problem is the idea that in humans, like in many other species, competition is individual, one-on-one, when in fact primates and humans frequently compete in teams or groups against other teams and groups [5]. Thus, the social context of competitions and their outcomes needs to be taken into account to arrive at a better understanding of the role of hormones in competitive encounters. This also requires the consideration of other motivational factors and hormones, such as those related to in-group affiliation [6] in competing teams. A third problem is the predominantly male focus of traditional research on dominance and hormones in humans: many studies look only at men and their T responses to competitions or, when women were included, still focused on T as the presumed "dominance hormone". However, research on human and non-human primates suggests that E may be the more important hormone for female dominance [7], [8]. In the present research, we addressed these issues in a computer-based competition paradigm with an experimentally varied outcome (win, loss) that allowed us to test men and women in individual and team competitions against another individual or team of their own gender. We studied effects of participant gender, contest outcome, single/team status, and participants' motivational needs for power and affiliation on changes in salivary T, E, P, C, and aggression to address the following questions: (1) Do experimental factors have a strong direct effect on changes in dominance-related hormones and aggression or do their interactions with dispositional motivational needs have to be taken into account? (2) Is there a difference in aggression and hormonal responses to dominance contests depending on whether one competes as an individual or as a team? (3) Are men's and women's hormonal and aggressive responses to dominance contests differentially related to T and E? (4) What role, if any, does the need for affiliation play in team competitions and hormonal and aggressive responses to the outcome?

### Hormones and competition

In humans, T fluctuations are thought to result from gaining or losing status within a dominance hierarchy [9]. Mazur’s [10] influential biosocial model of status predicts that in one-on-one competitions between males, gaining status (a win) is associated with a T increase and losing status (a loss) is associated with a T decrease, mediated by a stress-induced C increase. The post-contest T difference between winners and losers has been termed the *winner/loser effect* [9], [10]. Another influential model of the relationship between T and dominance–Wingfield et al’s [2] *challenge hypothesis*–similarly assumes that T changes are elicited by male one-on-one encounters. The challenge hypothesis is based on observations in male birds, which show heightened T during breeding season, further rising T in response to dominance contests with male conspecifics, and an increase of aggression associated with this socially elicited T increase.

These animal-based models of the relationship between T, dominance contests, and social behavior (including aggression) have been applied to humans with the expectation that in this species, too, T should rise in dominance contests, particularly after a victory, but decline after a defeat, and that T changes should be associated with increases or decreases, respectively, in aggressive behavior aimed at establishing and maintaining dominance (e.g., [11]; for overviews, see [12], [13], [14], [15], [9]). However, studies testing these hypotheses have yielded inconsistent findings. For instance, Hamilton et al [15] report that only 47% of the studies they reviewed show a clear winner/loser effect, with T elevated in winners of dominance contests and lowered in losers, whereas 49% of studies fail to find such an effect and the remaining 4% even report a reversed winner/loser effect. Other recent reviews corroborate the results of their review [13], [3]. Likewise, in a review of studies focusing on the link between increasing T and aggression, Archer [12] concluded that there is no consistent evidence for such an association.

Why do human studies applying biosocial models of dominance, T, and aggression yield such inconsistent results? We suggest that several factors are at work. With regard to T and aggression, the use of self-report measures in research with humans may have obscured existing associations between T and aggression. This methodological shortcoming was criticized by Mazur and Booth [9] and also by Archer [12]. Indeed, recent studies using behavioral testing paradigms show more promise in documenting links between T and aggressive behavior (for a review, see [13]).

Another factor is the application of models that have been developed based on face-to-face encounters in *individual* animals to human competitions with more complex social dynamics, such as team competitions in sports. While such an extension of the original model may better accommodate group-based competition characteristic of primates [5], hormonal responses to competition between male groups have been shown to differ from those of male individuals in one-on-one contests ([16–18]). This suggests that in men, the winner/loser effect is more likely to occur in one-on-one competitions, as predicted by the original biosocial model, than in team competitions, which may feature hormonal dynamics not covered by the biosocial model. For instance, it seems plausible to expect that social support of team mates to some extent buffers or inhibits the stressful C increase associated with dominance stress (e.g., [19, 20]). One consequence of this social buffering effect may be that losers may not show a decrease in T, otherwise elicited by increased C [21], and the winner/loser difference effect in post-contest T may therefore not be detected.

Yet another factor may be the application of models that have been developed for predicting males’ hormonal responses to dominance contests to females. Mazur and Booth [9] expected the biosocial model of dominance and T to hold for men, but not for women. And in support of this prediction, Carré and Olmstead [13] observed that the winner/loser effect occurs even less frequently in women than in men. However, “less frequently” cannot be equated with “not at all”, and there is clear-cut evidence from some sports competition studies that women respond with increased T to competitions and even show the winner/loser effect [14]. Interestingly, some of the strongest and most clear-cut winner/loser effects for T in women have been found for team competitions [22, 23]. This may suggest that in contrast to the case of male dominance contests, women are more likely to show a winner/loser effect in team competitions than in one-on-one contests, although the mechanism behind such an effect is even less well understood for women than it is for men. Some evidence indicates that T in women promotes bonding and affiliation in a team context ([24–26]), but may also be related to status seeking among teammates [25, 27].

In addition, there is also evidence that T may not be the focal hormone of female dominance seeking. Research on non-human primates ([8, 28]) and on humans ([7, 29]) suggests that E may play a key role in female intrasexual dominance competition. But as research by Stanton and Schultheiss [29] has shown, the link between E and women’s responses to dominance competitions may critically depend on individual differences in motivational dispositions, an issue that we turn to next.

### The moderating role of implicit motives

A growing number of studies suggests that in humans, individual differences in implicit (i.e., not reflected in individuals’ self-attributed motivational needs or explicit intentions [30, 31]) motivational needs moderate the effects of situational factors on hormonal changes [4]. Schultheiss and colleagues reported that in men, higher levels of nPower, defined as a capacity for drawing pleasure from one's impact on others, are associated with increasing T in response to winning and decreasing T in response to losing ([32–34]). In women, nPower predicts a similar pattern with regard to E, with rising E in response to winning and falling E in response to losing ([29, 35]). But in women, nPower also predicts general T increases in response to competing against another woman, regardless of winning or losing [34]. In both genders, nPower predicts increased C in response to dominance challenges and defeats and decreased C in response to victory ([36, 37]; for a full discussion of the functional and empirical links between dominance challenges, victory, defeat, stress hormones and gonadal steroid changes, see [38, 39]). Another line of research has implicated P in nAffiliation, a capacity for deriving pleasure from friendly, harmonious relationships. For instance, Schultheiss, Dargel, and Rohde [40] observed a positive association between P and n Affiliation in women not taking oral contraceptives, but a negative association between these variables in men. Wirth and Schultheiss [41] reported that increases in nAffiliation are associated with increases in P in bother genders. Brown et al [42] observed rises in P in situations in which people opened up to each other. Wirth [6] has argued that affiliation-associated P surges help to down-regulate stress responses and thus represent an endocrine basis for the stress-buffering effects of social closeness in general and perhaps of the female tend-and-befriend strategy [43] in particular. So far, however, the effect of dominance outcomes has been studied on nPower-dependent T, E, and C, but not on P; no study has examined the role of nAffiliation in the context of individual or team dominance contests; and the role of motives in hormonal changes due to team competitions has not been studied at all.

Beyond hormones, research has also documented that individuals high in nPower engage in aggressive behavior that is instrumental for promoting one’s impact on others or preventing others’ impact on oneself [44–46]. In general, research on nAffiliation suggests de-escalating effects of this motive on aggressive behavior in dominance conflicts and beneficial effects on coalition-building [47], although affiliation-motivated individuals can become aggressive in situations in which their relationships with others are at stake [46, 48].

### The present research

To summarize: In humans, winner/loser effects on T responses to a dominance contest appear to depend on more factors than contest outcome. First of all, there are gendered responses to individual versus team competitions. Further factors are motivational need dispositions related to dominance and social closeness and their interaction with situational variables. In addition, other hormones may play a critical role, such as E as a potential female dominance hormone, C as an indicator of individuals’ stress level and inhibitor of T release, and P as a hormone that is responsive to social affiliation, such as in teams, and may help down-regulate stress responses. Finally, observing an association between dominance-related T changes and aggression in humans may critically depend on the use of behavioral measures of aggression.

Usually, these issues are tested in separate studies, often with small sample sizes, with different assessment methods, and frequently in different laboratories. This makes it challenging to compare and integrate findings across multiple publications, to the extent that findings are even published [49]. In our research, we attempted to address these issues in one large-scale study, with consistent assessment methods across all conditions and a sufficiently large sample size to provide meaningful insights into the psychological, hormonal, and behavioral dynamics of dominance contests in humans. In so doing, we adapted a dominance contest paradigm described by Schultheiss et al [34] and combined it with the Taylor [50] aggression paradigm, thus turning aggressive behavior into a means to the end of dominance. Individual participants or pairs of participants (i.e., teams in the sense of a shared goal of beating the other team and high task interdependency in achieving this outcome; see [51]) were led to believe that they competed against other individuals or teams, respectively, for 10 rounds on a stimulus-response task on the computer. Participants were kept unaware that their opponents existed only as prerecorded video feeds presented on the computer screen. Feedback after each round (win or loss) was experimentally varied such that designated winners won, and losers lost, 8 out of 10 rounds. Before the beginning of each round, participants could adjust the volume of a noise blast their opponent(s) would receive upon losing the round. This represented a well-validated measure of their aggression [52]. Participants also received noise blasts over headphones whenever they lost a round, thus becoming the object of another (virtual) person's or team’s aggression. Participants provided saliva samples both before and after the contest while they also reported on their current mood. Samples were later assayed for T, E, P, and C. Participants' levels of nPower and nAffiliation were assessed via content-coding of a picture-story exercise administered before the contest.

### Hypotheses

Focusing first on effects due to gender or experimentally manipulated contest outcome and single/team status, we predicted the following patterns of results:

1 To the extent that our contest outcome manipulation works as intended, we expected wins and losses to lead to corresponding increases and decreases, respectively, in hedonic responses on a self-report mood scale, regardless of single/team competition status or gender.
2 We expected men to show a winner/loser effect on T after one-on-one contests and women to show a winner/loser effect on T, and possibly also E, in the team contest condition.
3 Consistent with a basic tenet of Mazur’s [1, 10] biosocial model of dominance, we expected to find an inverse winner/loser effect for C (i.e., higher post-contest C in losers than in winners), again with the prediction that this effect will be more likely to be observed for men in the one-on-one competition condition and for women in the team competition condition. We also speculated that team competitions would elicit overall weaker C responses, due to the social buffering of stress in an affiliative team context.
4 With regard to the relationship between T (or, in women, E) and aggression, we expected increases in these hormones to be associated with increased behavioral aggression across the contest.

With regard to moderating effects of implicit motives on effects of gender and experimentally manipulated factors on hormones and behavior, we made the following predictions:

5 For the one-on-one contests, we expected to replicate the nPower x Contest Outcome effects on T changes reported by Schultheiss et al [34]; that is, we predicted that nPower would be associated with post-contest T increases in male and female winners and with post-contest T decreases in male losers (Schultheiss et al. [34] did not report a negative association for women).
6 For the one-on-one contests, we also expected to replicate the nPower x Contest Outcome effects on E changes reported by Stanton and Schultheiss [29]; that is, we predicted that nPower would be associated with post-contest E increases in female winners and with post-contest E decreases in female losers.
7 For the one-on-one contests, we expected to replicate the nPower x Contest Outcome effects on C changes for women and men reported by Wirth et al [37]; that is, we predicted that nPower would be associated with post-contest C increases in losers and with post-contest C decreases in winners.(Analyses regarding the team contest condition were exploratory with regard to hypotheses 4 through 7, but with an expectation of similar effects.)
8 For team competitions, we expected to see an association between nAffiliation and post-contest P increases, and likely more so in women, for whom this association is well-documented, than in men, for whom inconsistent findings exist [4, 40]. For one-on-one competitions, we predicted no association between nAffiliation and P due to the absence of an ally participants could associate with.
9 We expected nPower to predict increases and nAffiliation to predict decreases in aggression over the course of the contest, consistent with the self-enhancing versus relationship-building functions of these motives, respectively [4].

For hormones under the control of the hypothalamic-pituitary-gonadal axis (i.e., T, E, and P) we additionally explored effects of oral contraceptives (OC), which inhibit the normal function of this axis, on our predicted findings. We expected a greater likelihood to observe predicted effects on gonadal hormones for normally cycling (NC) women, provided that effects depend on normal gonadal function. Alternatively, an absence of inhibiting OC effects may point to the adrenals, which also produce P and androgens, as the source of hormone changes [53].

## Method

### Participants

Participants were recruited from the campus of Friedrich-Alexander University, Erlangen, Germany, and from online message boards. We obtained written informed consent from all participants. After exclusion of three participants who had completed only the picture-story measure of implicit motives but no other task and of five participants who had not completed the full picture-story measure, the resulting final sample consisted of 326 participants (mean age: 21 years), of which 165 were male and 161 were female. Of the latter, 96 reported current OC use. Ninety-three percent of the sample were of German nationality, and all participants were fluent in German. Sessions lasted around 2 hours and participants were paid 20 €. Diurnal variations in hormone levels were minimized by only having sessions start after 1:30 pm. No psychology majors participated. This study was approved by the Institutional Review Board of Friedrich-Alexander University.

### Study design

The study consisted of same-sex one-on-one and two-on-two matches (Condition) separately for men and women (Sex; analyses for which oral contraceptive use was relevant used the factor Group instead, consisting of men, NC women, and OC women). Participants were randomly assigned to win or lose (Contest Outcome) and one-on-one or two-on-two conditions. The competition consisted of a 10-round contest based on a serial response task [34] combined with an opportunity to aggress against an opponent by applying noise blasts [50] in the context of a virtual competition. Dependent variables were changes in salivary T, E, P, and C, and aggression, as assessed via noise blast settings entered at the beginning of each contest round.

### Procedure

All data were collected on computers and programmed in Inquisit 4 (Millisecond Software; Seattle, WA, USA). All sessions were run by a single experimenter and consisted of a pre-contest phase, a contest lasting 15 min, and a post-contest phase. One-on-one and two-on-two matches differed only during the contest. In the pre-contest phase, participants completed a motive measure, a mood questionnaire, and other tasks unrelated to the findings reported here. The pre-match saliva sample was then taken at the end of the pre-contest phase, immediately before participants entered the contest. For the one-on-one contest, the computer program (https://osf.io/9fg8a/) informed participants that they would compete on a serial response task against the next available opponent, provided instructions for the competition, and asked them to enter their first name. The justification for the contest provided to participants in all conditions stated that performance is often enhanced if people compete on a task rather than working on it by themselves and that therefore participants would perform the next task as a contest. The computer script then presented participants with a pre-recorded video of their opponent (same gender as participant), introduced them by name, and asked them to wave to each other. This was intended to embellish the illusion of being part of a live interaction with another person. Next, the script introduced the punishment paradigm, instructing participants before each round to set the level of a noise-blast punishment that would be given to their (virtual) opponent should they win the round. This was intended to introduce aggression as a power incentive, that is, as means to have impact on one’s opponent, and at the same time to avoid a power disincentive, that is, an opponent’s impact on oneself, should she or he win the round. The punishment component was justified to participants by referring to a motivation-enhancing effect of being able to deliver punishment to one's opponent after winning a given round. Participants listened via headphones to examples of blasts of white noise at 25%, 50%, 75% and 100% volume (max 85 dbs). At the beginning of each of 10 rounds of the subsequent contest, participants could use a slider to set the noise-blast punishment volume between 0% and 100%, with 50% preset as a default. After a 5-sec countdown, a round of the serial response task ensued, requiring participants to respond as quickly and accurately as possible to stimuli appearing on 4 different screen locations by pressing the corresponding 4 keys on a key pad. After 60 s, the task stopped and the screen showed the word “Calculating”. For winning trials, the next screen showed the words "You have won this round" on a green background, accompanied by a jubilant jingle, and presented a prerecorded video of the opponent(s) receiving a noise blast. For losing trials, the next screen showed the words "You have lost this round" on a red background and participants received a white-noise blast. In this case, a prerecorded video created the impression that their opponent watched them while they received the blast. The sequence of winning and losing was fixed, such that in the win (lose) condition participants won (lost) every round except for the second and the fifth. Similarly, the noise-blast intensity received by participants was fixed. In the win condition, participants received two noise blasts, one each after the two rounds they lost (noise blast sequence, in % of maximum setting, with “-”denoting absent noise blasts: -, 80, -, -, 100, -, -, -, -, -). In the lose condition, participants received 8 noise blasts, one each after the 8 rounds they lost (noise blast sequence: 60, -, 70, 80, -, 100, 90, 90, 90, 90, 90). In the two-on-two contest, participants were paired with another participant of their own gender and worked as a team on during the contest. This entailed splitting the serial response task such that each participant was responsible for stimuli appearing in her or his respective half of the screen and watching prerecorded videos of teams of opponents (same gender as participants) instead of individual opponents throughout the contest. At the conclusion of the match, participants were led to their separate cabins again.

During the post-contest phase participants collected two more saliva samples, with Sample 2 taken immediately after the contest (i.e., 20 min after Sample 1) and Sample 3 taken 20 min later. We thereby aimed to capture early hormonal responses (Sample 2) as well as the “sweet spot” of salivary hormonal responses to a psychologically relevant event occurring approximately 20 min post-event [54, 55]. Participants provided both samples while working on repeated presentations of the mood questionnaire. Towards the end of the post-contest session, which also contained some tasks unrelated to the findings presented here, participants completed the mood questionnaire a fourth time, provided biographic data, and completed a manipulation check in the form of two open-ended questions asking for anything they had noticed during the competition. As a final manipulation check the experimenter then asked them if they had noticed anything or if anything unusual had happened during the experiment. Participants were paid and debriefed about the study's hypotheses, the manipulation of the contest outcome, and the prerecorded videos of the opponent(s).

### Implicit motives

nPower and nAffiliation were assessed with a Picture Story Exercise (PSE), using standard instructions specified by Schultheiss and Pang [56]. Participants were given 5 min to write creative stories in response to pictures. Six pictures were chosen for the PSE stories: women in laboratory, ship captain, boxer, protester hurling a stone, bicycle race (for further description of these pictures, see [56]), and a novel picture showing two football teams facing each other. PSE stories were coded for nPower and nAffiliation in accordance with Winter's [57] Manual for Scoring Motive Imagery in Running Text by four trained coders, one each for male and female one-on-one and two-on-two conditions. nPower was coded for imagery related to (a) strong, forceful actions that have impact on others, (b) controlling or manipulating others, (c) influencing or persuading others, (d) offering unsolicited help or advice, (e) impressing others or showing a concern about fame, prestige, reputation, and (f) actions that elicit a strong emotional response in others. nAffiliation was scored whenever a character (a) expresses positive, intimate feelings towards others, (b) shows sadness about a separation, (c) engages in companionate activities, or (d) acts in a nurturing manner. The trained coders had reached 85% agreement with expert coding in the Winter Manual training materials. In addition, we ascertained within-study interrater reliability by having each coder blindly code at least 60 stories that had also been coded by another coder and making sure that interrater reliability, as estimated by Lin’s [58] coefficient of concordance was acceptable (i.e., > .70). On average (± *SD*), participants' six PSE stories featured 614 ± 171 words and contained a total of 8.54 ± 3.87 power and 2.54 ± 2.68 affiliation images. To correct for skew, motive scores were square-root-transformed after the addition of the constant 1. However, as there was evidence of between-coder bias and PSE word count was significantly correlated with nPower (*r*s = .46 to .60, *p*s < .00005) and nAffiliation scores (*r*s = .39 to .48, *p*s < .0005), we residualized scores for word count and converted these residuals to z scores separately for each of the four subsamples resulting from the Sex x Condition factorial (see [56]).

### Salivary hormones

For each of the salivary samples, participants collected up to 7.5 mL unstimulated saliva in a sterile polypropylene vial. Participants sealed the vials immediately after each collection. The experimenter placed the vials in frozen storage after each experimental session. Samples were freed from mucopolysaccharides and other residuals by three freeze–thaw cycles, followed by centrifugation. All standards and controls were diluted with water (resulting analytic ranges: 5–400 pg/mL for T; 0.51–19.50 pg/mL for E; 5–400 pg/mL for P; 0.5–25 ng/mL for C). Salivary hormones were determined by solid-phase 125-I radioimmunoassays (for T, E, and C: Coat-A-Count, Siemens Healthcare; for P: Progesterone RIA, BeckmanCoulter), using assay protocols previously validated for use with saliva [37, 40]. Lower limits of detection (B_0_—3xSD) were 3.02 pg/ml for T, 0.60 pg/ml for E, 1.04 pg/ml for P, and 0.06 ng/ml for C. Low and high control samples were included in each assay (BioRad Lyphochek; 20 and 100 pg/ml for T; 1.23 and 3 pg/ml for E; 27 and 101 ng/ml for P; 1.5 and 3.5 ng/ml for C). Average recovery values for low and high controls were 107% and 94% for T; 128% and 131% for E; 54% and 72% for P; 89% and 88% for C. Median intra-assay CVs were 10.94% for T; 21.86% for E; 8.91% for P; 4.59% for C. Due to low or missing volume in some samples, *n*s for salivary hormone assays were < 326. Visual histogram inspection and Shapiro-Wilks tests indicated that hormone values deviated from normal distributions. We therefore subjected P, E, and C to log transformations and T to a square-root transformation, after adding a constant of 1, and used these transformed scores in all inferential statistical procedures. Descriptive statistics are given for untransformed values.

### Aggression

Aggression was assessed as the slider setting (in % of maximum 85 db noise blast) participants chose at the beginning of each round for the punishment to be administered to their (virtual) opponents in case participants won the round. It could vary between 0% and 100%, in increments of 1%.

### Mood

We assessed participants' mood with the hedonic-tone scale of the mood measure developed by Matthews, Jones, and Chamberlain [59]. The scale is designed to assess states of motivational gratification and frustration and is comprised of the items *happy*, *satisfied*, *contented*, *cheerful*, *sad*, *depressed*, *dissatisfied*, and *sorry*. Items were presented in random order with the primer “Right now I feel…” and participants could endorse each item on a 4-point scale featuring the gradations *definitely not*, *slightly not*, *slightly*, and *definitely*. After recoding negatively keyed items, hedonic tone sum scores were calculated for each assessment. Coefficient alphas of the hedonic tone scale were .79, .90, .85, and .87 for T1 through T4.

### Analyses

All analyses were performed with SYSTAT 13. For analyses of mood and hormone changes, we used repeated-measures ANCOVA, with Time (T2 and T3) as within-subjects factor, mood/hormone at T1 (baseline) as covariate, and Sex, Contest Outcome, and Condition as between-subject factors. In subsequent analyses of individual differences in implicit motivational needs, we reran the same models for hormones with the inclusion of either nPower or nAffiliation scores and their interactions with categorical factors and included the respective covariates in all follow-up analyses. Significant interactions from these regression analyses were followed up with simple slope analyses. When Time was not a significant moderator of an effect on hormones, we averaged post-contest hormone values (i.e., T2 and T3) and dropped time as a within-subjects factor from the analysis. Whenever we found oral contraceptives to make a significant difference for women's hormonal responses, we replaced the variable Sex with the variable Group (OC, NC, or male) and report analyses including this latter variable. All *η*^*2*^ effect size estimates represent partial effects.

## Results

### Manipulation and suspicion checks

**Fig 1:**
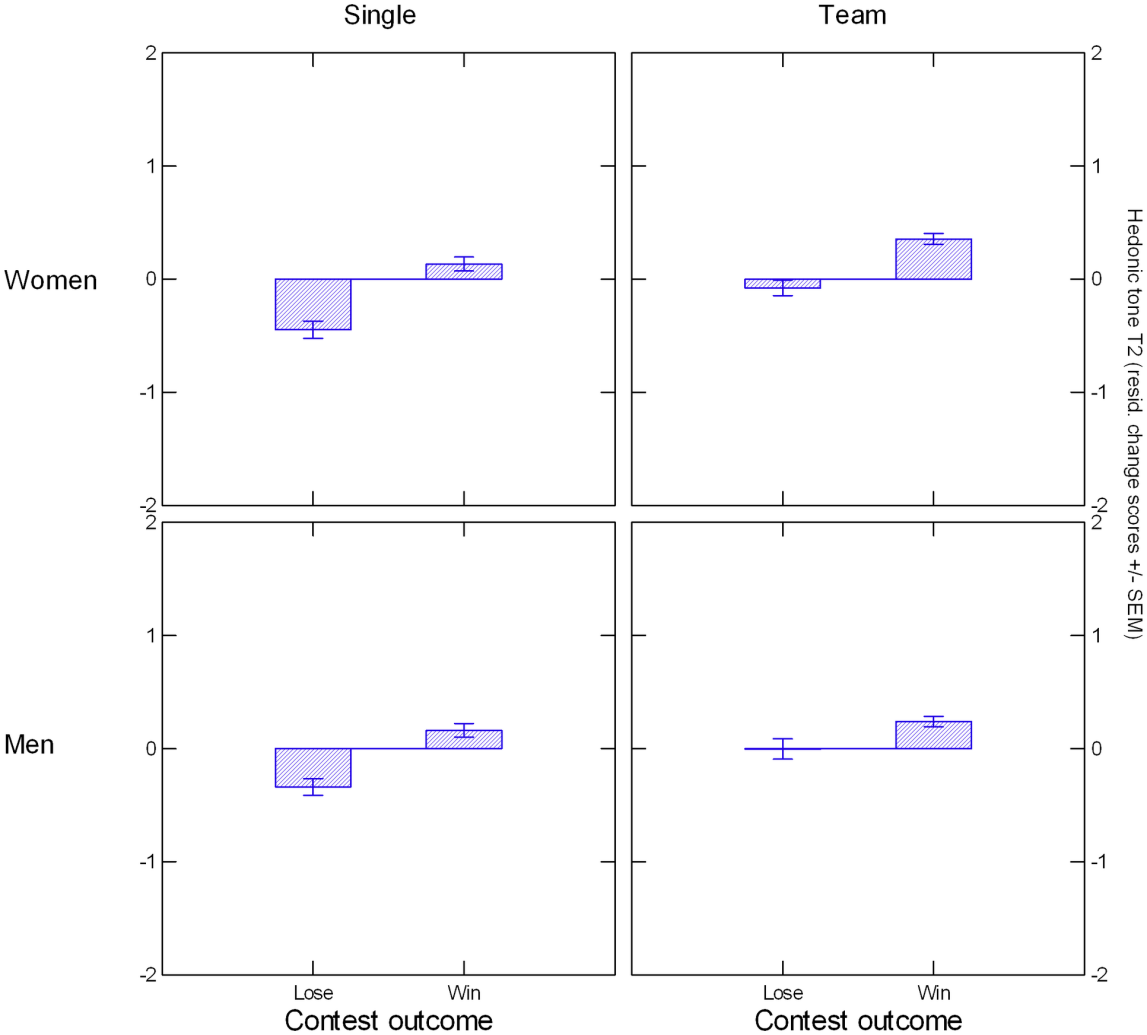
Contest outcome effect on changes in hedonic tone, immediately post-contest (T2) and holding hedonic tone at T1 constant, plotted separately for all 4 subsamples.

T1 hedonic tone did not differ as a function of Contest Outcome, *F*(1, 323) < 1. As a manipulation check we examined the effect of Contest Outcome on hedonic tone at T2 controlling for T1 and found that winning and losing affected changes in hedonic tone, *F*(1, 322) = 80.72, *p <* 0.000005, *η*^*2*^ = .200. Further, we found changes in hedonic tone to be associated with Outcome in each of the subsamples resulting from the Sex x Condition factorial: Women single, *F*(1, 78) = 42.95, *p <* 0.000005, *η*^*2*^ = .355; men single, *F*(1, 83) = 26.48, *p* = 0.000002, *η*^*2*^ = .242; women team, *F*(1, 77) = 28.09, *p* = 0.000001, *η*^*2*^ = .267; men team, *F*(1, 75) = 7.42, *p* = 0.008, *η*^*2*^ = .090. Fig 1 shows that, for both men and women and in both conditions, hedonic tone at T2 increased for winners and decreased for losers. We conclude from these findings that the contest manipulation worked in terms of eliciting affective responses that were consistent with participants’ contest outcome.

Of the 326 participants tested, 25 (7.67%) indicated on the open-ended question that they had doubts about the veracity of the contest outcome and/or the authenticity of the opponent(s). Fourteen participants (4.52%) volunteered comments of this nature to the experimenter upon session completion. In total, 31 participants (9.51%) voiced doubts about the contest in writing and/or verbally at the end of the data collection session. To examine whether these doubts had an undermining effect on participants' previous subjective experience of winning or losing the contest, we examined whether voicing doubts moderated the effect of contest outcome on hedonic tone at T2, controlling for T1 levels. The effect was not significant (*p* = .88), and the slopes for hedonic tone residuals were almost identical for participants voicing doubts (*B* = 0.401, *SE* = 0.202, *t*(28) = 1.98, *p* = .06) and those who did not (*B* = 0.436, *SE* = 0.050, *t*(291) = 8.73, *p <* .0000005). We conclude from these findings that whatever suspicions some participants voiced at the end of data collection was not sufficient to substantially change the immediate affective impact of the contest and its outcome. Consistent with this, whether participants were suspicious or not at the end of the experiment did not moderate the findings reported in the following, nor did exclusion of suspicious participants alter them.

### Testosterone

**Fig 2:**
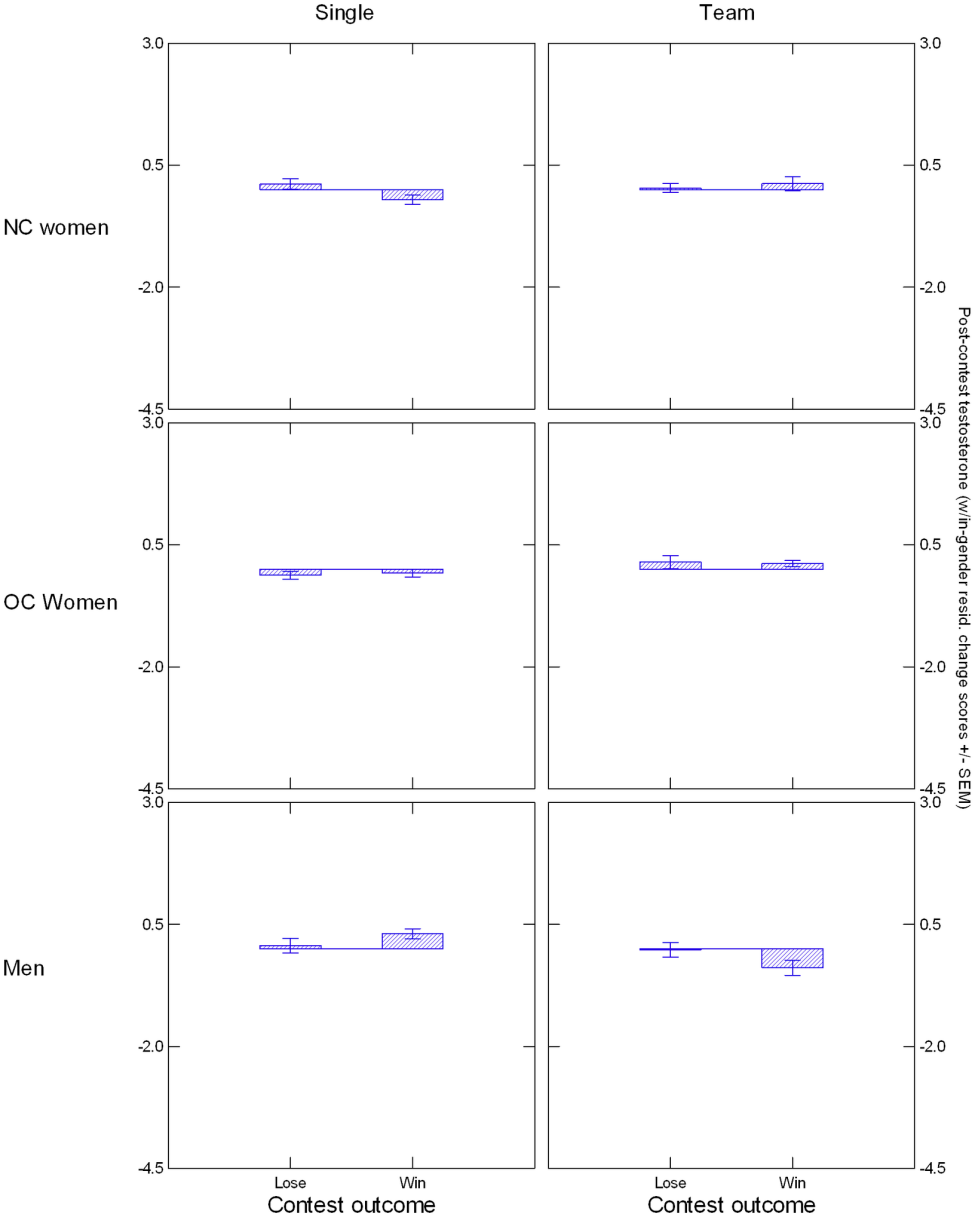
Illustration of Condition x Outcome x Group interaction. Post-contest testosterone (averaged across square-root-transformed T2 and T3 values and residualized within each group for square-root-transformed T1 values) are presented on the Y axis.

Table 1 provides descriptive statistics for participants’ salivary T at T1 through T3, with all measures showing a declining trend that most likely reflects circadian changes [60, 61] (see S1 File for within- and between-hormone correlations). OC and NC women had significantly different T values at each assessment, *t*s > 4.93, *p*s < .000002. A repeated-measures ANCOVA focusing on the between-subjects factors Condition, Contest Outcome, and Group with averaged T at T2 and T3 as dependent variable and T at T1 as covariate yielded an Outcome x Condition x Group interaction, *F*(2, 264) = 2.88, *p* = .058, *η*^*2*^ = .021, illustrated in Fig 2. Follow-up analyses indicated that the interaction was driven by a significant Outcome x Condition effect in men, *F*(1, 147) = 3.91, *p* = .050, *η*^*2*^ = .026, and NC women, *F*(1, 46) = 4.66, *p* = .036, *η*^*2*^ = .092, that did not emerge in OC women, *p* > .10. In men, the interaction was due to winning individuals having higher residualized post-contest T than winning teams, *F*(1, 78) = 13.90, *p* = .0004, *η*^*2*^ = .151, but within each competition type, winners’ T changes did not significantly differ from losers’. NC women showed the opposite response pattern: Winning individuals had lower residualized post-contest T than winning teams, *F*(1, 22) = 4.73, *p* = .041, *η*^*2*^ = .177, and they also differed from losing individuals, who showed higher residualized post-contest T, *F*(1, 27) = 7.12, *p* = .013, *η*^*2*^ = .209.

**Table 1:** Descriptive statistics (*M* [*SD*, *n*]) for salivary hormone measurements.

	Time	Testosterone (pg/mL)	Estradiol (pg/mL)	Progesterone (pg/mL)	Cortisol (ng/mL)
Men	T1	78.95 (33.45, 152)	0.99 (0.60, 103)	16.27 (12.74, 144)	2.59 (1.31, 137)
	T2	74.92 (30.47, 152)	0.80 (0.56, 105)	10.73 (6.98, 145)	2.09 (1.01, 137)
	T3	72.42 (28.90, 152)	0.68 (0.48, 104)	8.66 (5.55, 142)	1.80 (0.82, 137)
Women (OC)	T1	9.10 (5.13, 77)	0.72 (0.75, 90)	16.59 (16.00, 85)	2.72 (1.13, 64)
	T2	7.34 (4.24, 79)	0.60 (0.63, 87)	12.30 (16.35, 85)	2.32 (0.90, 65)
	T3	6.68 (4.04, 78)	0.52 (0.60, 86)	10.60 (13.92, 80)	2.06 (0.75, 65)
Women (NC)	T1	14.58 (7.37, 53)	1.60 (1.06, 59)	37.41 (41.35, 57)	2.73 (1.45, 43)
	T2	12.23 (5.91, 54)	1.38 (1.00, 59)	33.75 (43.87, 62)	2.31 (1.17, 43)
	T3	11.80 (5.83, 53)	1.28 (0.94, 60)	31.44 (44.90, 58)	2.04 (1.06, 43)

**Fig 3:**
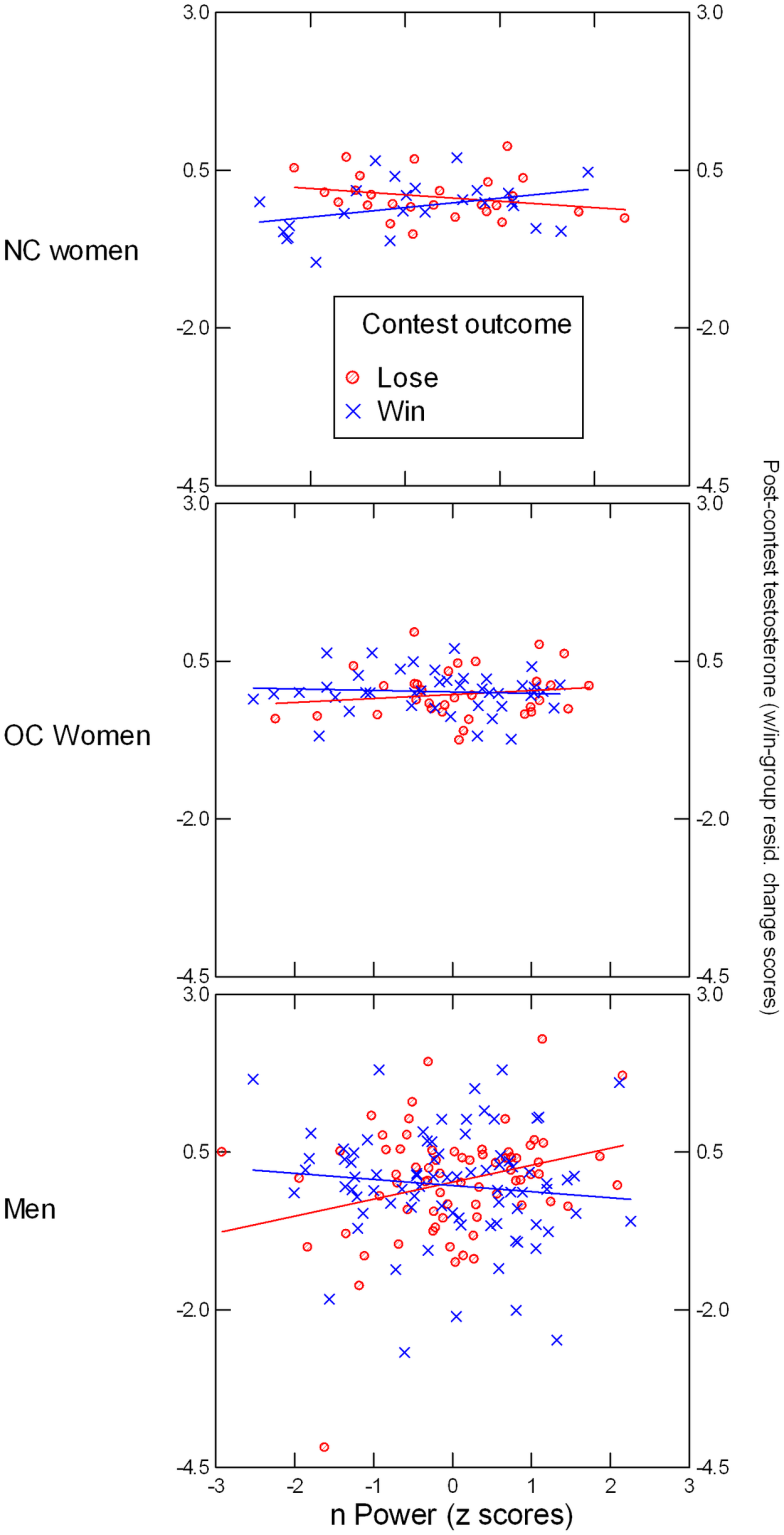
Illustration of nPower x Outcome x Group interaction. Post-contest testosterone (averaged across square-root transformed T2 and T3 values and residualized within each group for square-root transformed T1 values) are presented on the Y axis. Lines (blue = winners, red = losers) represent regression slopes.

Including nPower in the repeated-measures ANCOVA revealed a significant Group x Outcome x nPower interaction, *F*(2, 264) = 3.83, *p* = .023, *η*^*2*^ = .028, illustrated in Fig 3. Follow-up analyses indicated that the Outcome x nPower interaction was significant for men *F*(1, 147) = 6.22, *p* = .013, *η*^*2*^ = .041 and NC women *F*(1, 46) = 6.91, *p* = .012, *η*^*2*^ = .131, but not for OC women, *F*(1, 69) = 0.96, *p* = .33. Simple slope tests indicated that in men, the association between nPower and post-contest T was positive in losers, *B* = 0.269, *SE* = 0.109, *t*(68) = 2.48, *p* = .015, and negative in winners, *B* = -0.101, *SE* = 0.098, *t*(78) = -1.02, *p* = .31, and in NC women, it was negative in losers, *B* = -0.147, *SE* = 0.083, *t*(23) = -1.78, *p* = .088, and positive in winners, *B* = 0.171, *SE* = 0.085, *t*(22) = 2.00, *p* = .058. Analyses using nAffiliation instead of nPower did not yield significant main or interaction effects involving this variable.

### Estradiol

OC and NC women had significantly different E values at each assessment, *t*s > 6.12, ps < .0000005. A repeated-measures ANCOVA based on the factors Contest Outcome, Condition, and Group revealed a Group x Condition interaction on averaged post-contest E, *F*(2, 244) = 2.77, *p* = .064, *η*^*2*^ = .022. This effect was due to NC women in the single condition, who had significantly higher residualized post-contest E (0.66 ± 0.03) than OC women (0.52 ± 0.02) or men (0.51 ± 0.02) in the same condition or NC women in the team condition (0.54 ± 0.03), *p*s < .05. No other between-group differences became significant.

**Fig 4:**
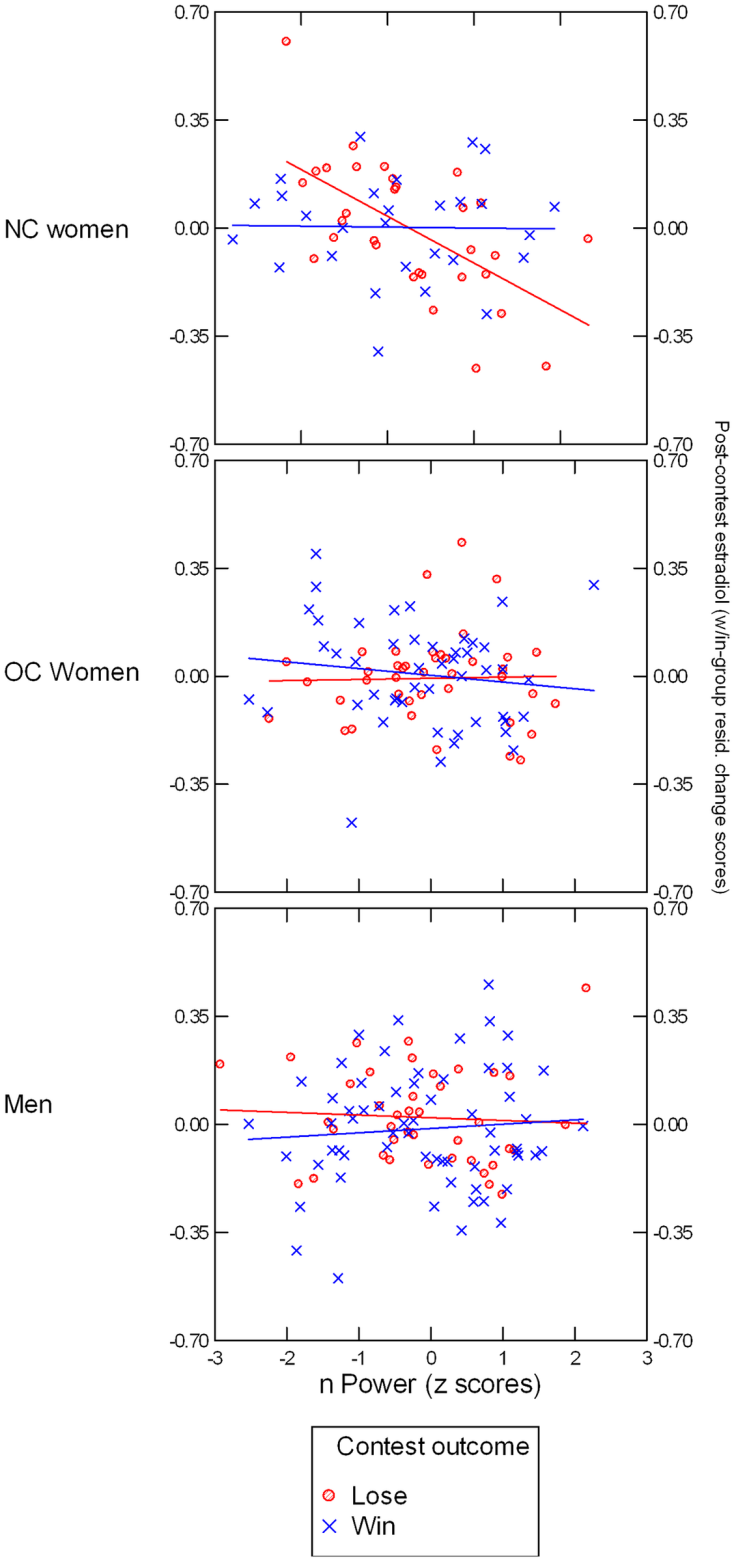
Illustration of nPower x Outcome x Group interaction. Post-contest estradiol (averaged across log-transformed T2 and T3 values and residualized within each group for log-transformed T1 values) are presented on the Y axis. Lines (blue = winners, red = losers) represent regression slopes.

Including nPower in the repeated-measures ANCOVA revealed a significant Group x Outcome x nPower interaction, *F*(2, 238) = 4.03, *p* = .02, *η*^*2*^ = .033 (see Fig 4). Follow-up analyses indicated that the three-way interaction was due to an Outcome x nPower effect in NC women *F*(1, 54) = 9.08, *p* = .004, *η*^*2*^ = .144, that did not emerge for OC women or men, *p*s > .46. Simple slope tests indicated that in NC women, the association between nPower and residualized post-contest E was nonsignificant in winners, B = -0.003, SE = 0.033, *t*(25) = -0.10, *p* = .93, and negative in losers, *B* = -0.151, *SE* = 0.038, *t*(28) = -4.04, *p* = .0004. Analyses using nAffiliation instead of nPower did not yield significant main or interaction effects involving this variable.

### Progesterone

OC and NC women had significantly different P values at each assessment, *t*s > 4.29, *p*s < .001. A repeated-measures ANCOVA based on the factors Contest Outcome, Condition, and Group only yielded a main effect of Group, *F*(2, 282) = 6.62, *p* = .002, *η*^*2*^ = .045. NC women had significantly higher post-contest residualized average P (2.54 ± 0.05) than OC women (2.35 ± 0.04) or men (2.32 ± 0.03), *p*s < .05.

**Fig 5:**
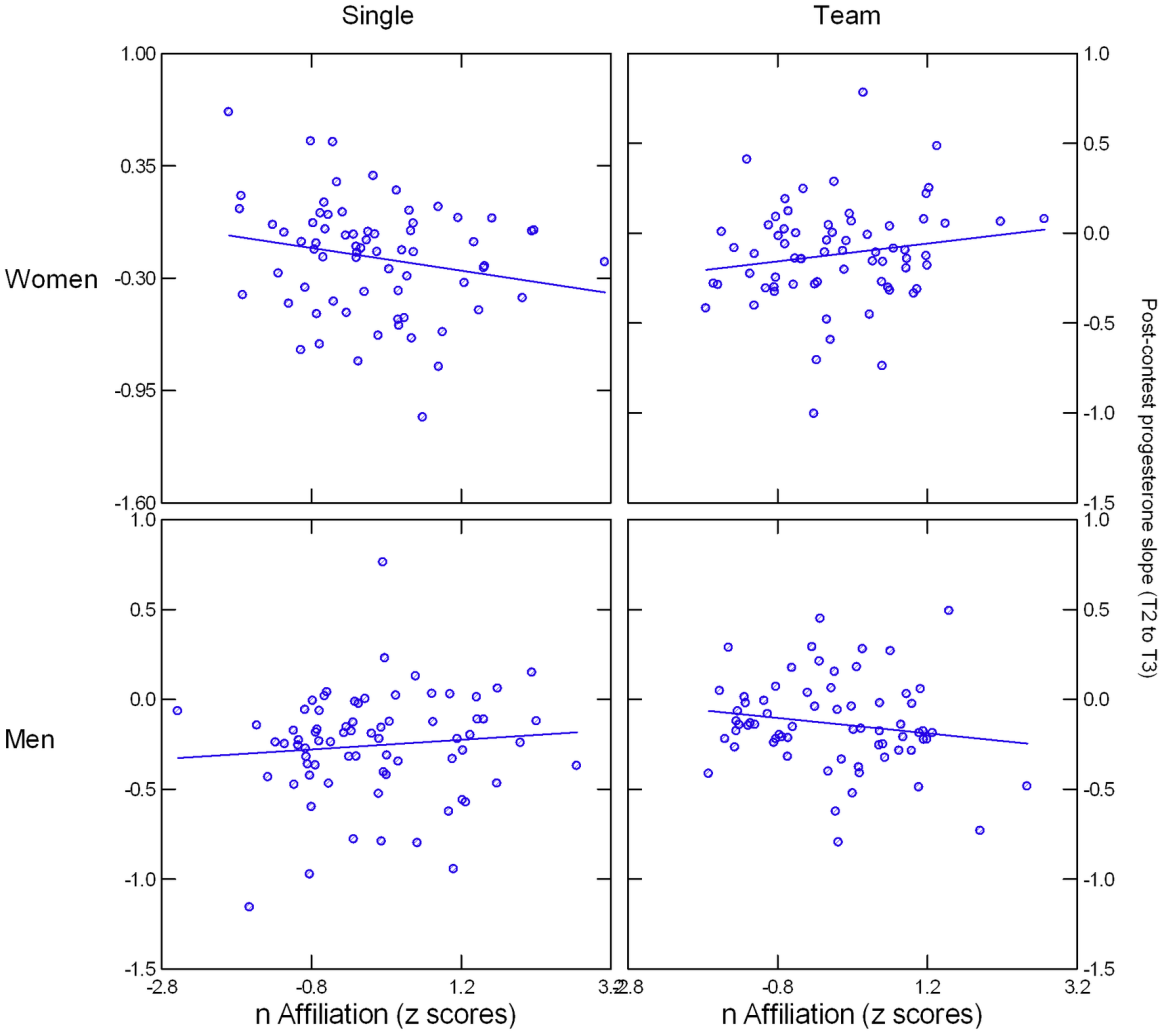
Illustration of Condition x Gender x nAffiliation x Time effect, with the data of 4 outliers removed. Y-axis values represent slopes from T2 to T3, with positive values indicating and increase and negative values a decrease from 0 to 20 min post-contest.

Including nAffiliation in the repeated-measures ANCOVA revealed a significant Group x Condition x nAffiliation x Time interaction, *F*(2, 259) = 3.12, *p* = .046, *η*^*2*^ = .034. The effect became more pronounced after removing 4 outliers with studentized residuals > |4.18| and replacing Group with Sex, suggesting that OC use among women was not responsible for the interaction effect, *F*(1, 259) = 9.64, *p* = .002, *η*^*2*^ = .036 (see Fig 5). The effect was due to a Condition x nAffiliation x Time interaction in women, *F*(1, 125) = 7.67, *p* = .006, *η*^*2*^ = .059, that did not emerge in men, *F*(1, 133) = 2.53, *p* = .11, *η*^*2*^ = .019. To represent the Time factor and simplify follow-up analyses, we calculated each participant's slope from T2 to T3, with positive values indicating an increase and negative values a decrease in P from immediately after the contest to 20 min later. nAffiliation predicted a negative P slope and hence a steeper decline, *B* = -0.078, *SE* = 0.039, *t*(63) = -2.00, *p* = .050, for women in the single-competition condition and a positive P slope and hence a steeper increase, *B* = 0.066, *SE* = 0.035, *t*(61) = 1.88, *p* = .065, for women in the team-competition condition. Analyses using nPower instead of nAffiliation did not yield significant main or interaction effects involving this variable.

### Cortisol

**Fig 6:**
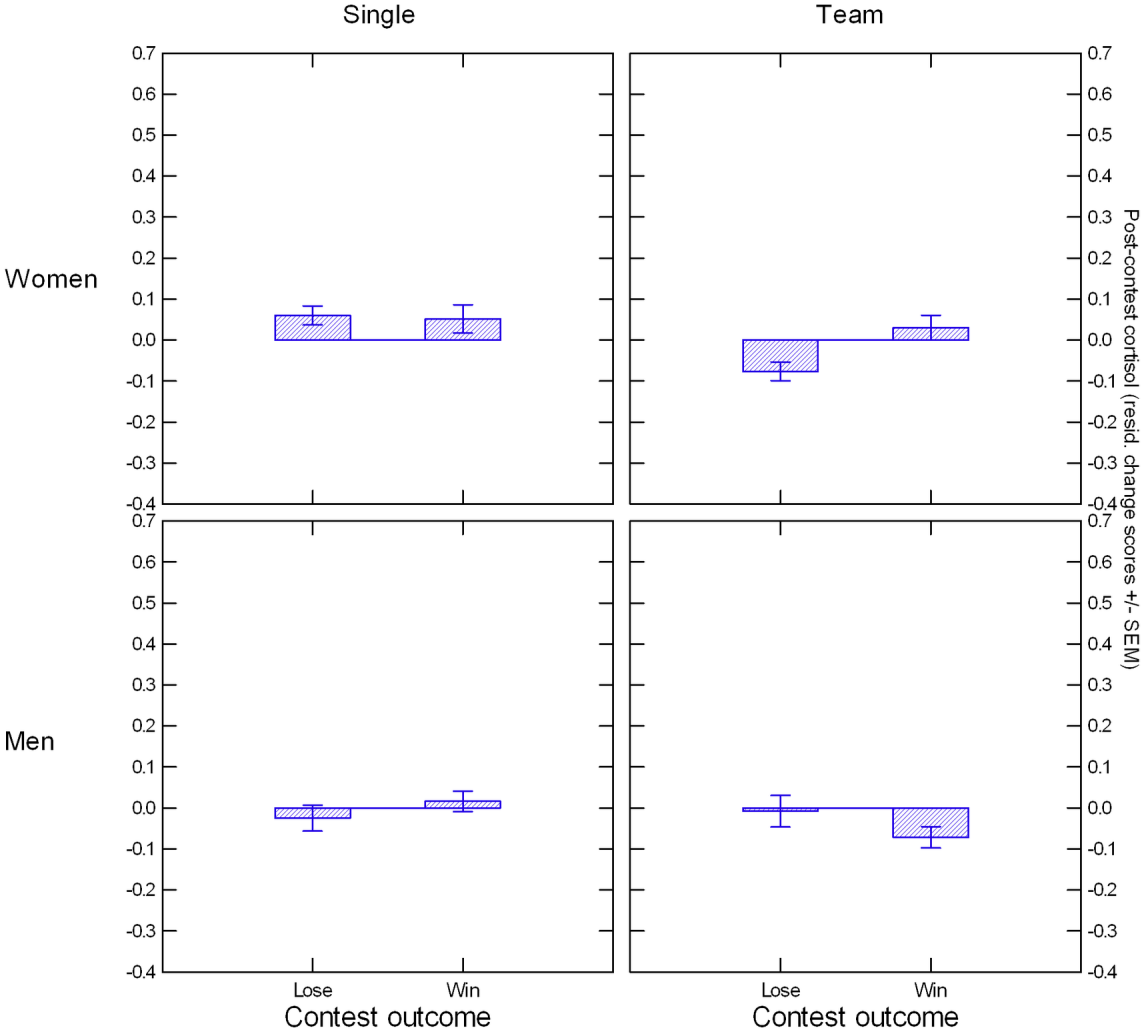
Illustration of Condition x Outcome x Gender effect. Post-contest cortisol (averaged across log-transformed T2 and T3 values and residualized for log-transformed T1 values) are presented on the Y axis.

OC and NC women did not significantly differ in their C levels, *t*s < 1, *p*s > .61. A repeated-measures ANCOVA focusing on the between-subjects factors Condition, Contest Outcome, and Sex with averaged C at T2 and T3 as dependent variable and C at T1 as covariate yielded an Outcome x Condition x Sex interaction, *F*(1, 235) = 6.35, *p* = .012, *η*^*2*^ = .026, illustrated in Fig 6. Follow-up analyses indicated that the three-way interaction was due to an Outcome x Sex effect in the team condition, *F*(1, 99) = 6.71, *p* = .011, *η*^*2*^ = .063. The two-way interaction in the team condition was primarily based on female teams having higher residualized post-contest C after winning than after losing, *F*(1, 37) = 5.75, *p* = .02, *η*^*2*^ = .134. In the single-competition condition, the Outcome x Sex effect did not emerge, *F*(1, 135) = 0.79, *p* = .38, *η*^*2*^ = .006. Instead, we obtained a main effect of gender, *F*(1, 139) = 4.32, *p* = .040, *η*^*2*^ = .031, with women showing stronger residualized post-contest C increases than men.

**Fig 7:**
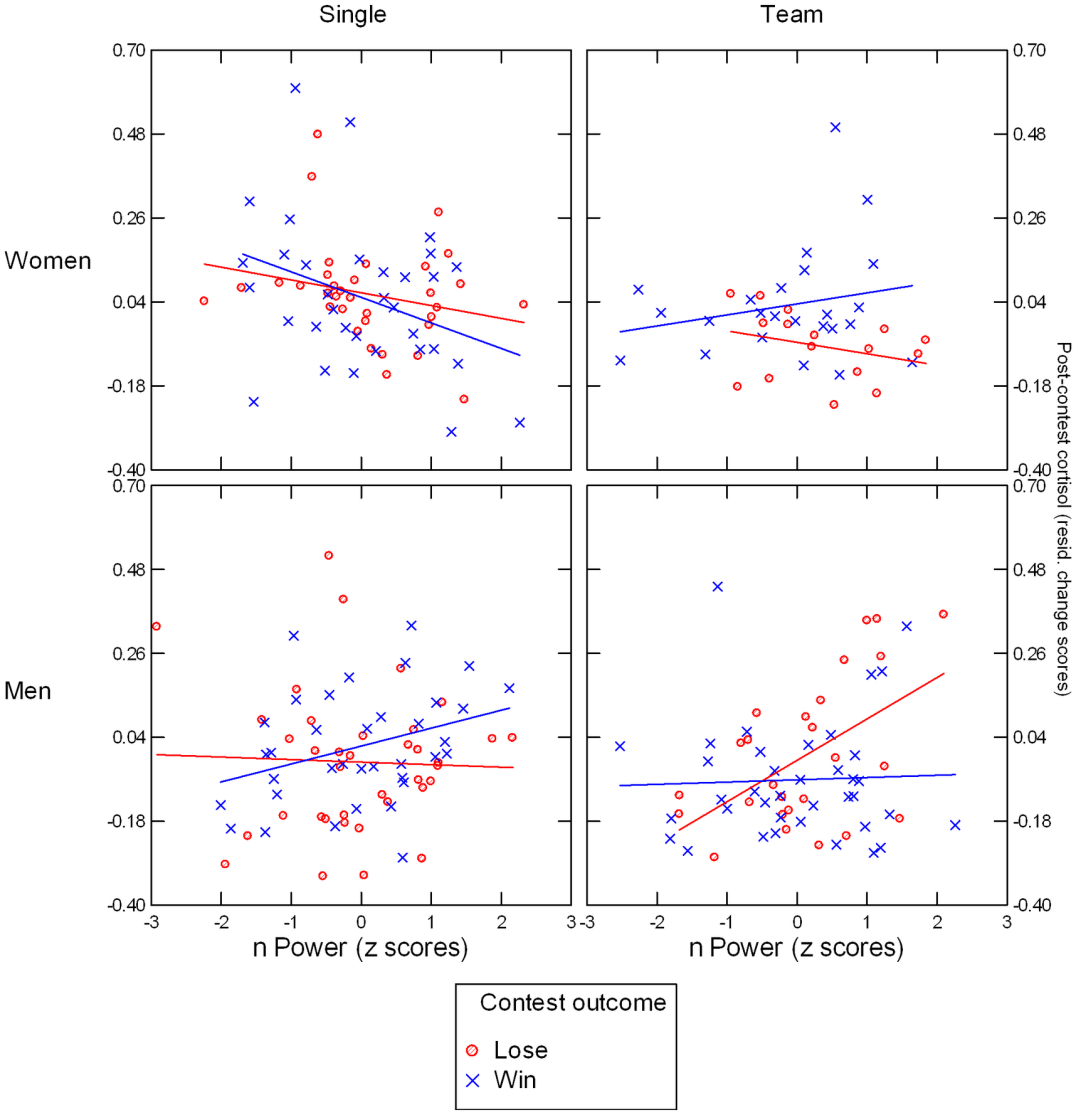
Illustration of nPower x Condition x Outcome x Gender effect. Y axis shows post-contest cortisol (averaged across log-transformed T2 and T3 values and residualized for log-transformed T1 values).

Including nPower in the repeated-measures ANCOVA revealed a significant Outcome x Sex x Condition x nPower interaction, *F*(1, 227) = 8.00, *p* = .005, *η*^2^ = .034, that was not moderated by Time (see Fig 7). Follow-up analyses revealed that this effect was due mainly to a Sex x Outcome x nPower interaction on averaged post-contest C as dependent variable in the team-competition condition, *F*(1, 95) = 6.06, *p* = .02, *η*^2^ = .060, which in turn was based on a significant Outcome x nPower effect in men, *F*(1, 59) = 5.21, *p* = .03, *η*^2^ = .081, but not in women, *F*(1, 35) = 1.48, *p* = .23, *η*^2^ = .040. For men in winning teams, nPower had no significant effect on residualized post-contest C, *B* = 0.011, *SE* = 0.027, *t*(35) = 0.41, *p* = .68, but for men in losing teams, it predicted higher residualized post-contest C, *B* = 0.109, *SE* = 0.036, *t*(23) = 3.00, *p* = .006. In the single-competition condition, the Sex x Outcome x nPower effect failed to become significant, *F*(1, 131) = 2.37, *p* = .13, *η*^2^ = .018. Instead, ANCOVA yielded a significant Sex x nPower effect in the single-competition condition, *F*(1, 135) = 6.32, *p* = .01, *η*^2^ = .045, which was based on a negative association between nPower and residualized post-contest C in individually competing women, *B* = -0.053, *SE* = 0.020, *t*(64) = -2.69, *p* = .009, that did not emerge for individually competing men, *p* = .27. Analyses using nAffiliation instead of nPower did not yield significant main or interaction effects involving this variable.

### Aggression

**Fig 8:**
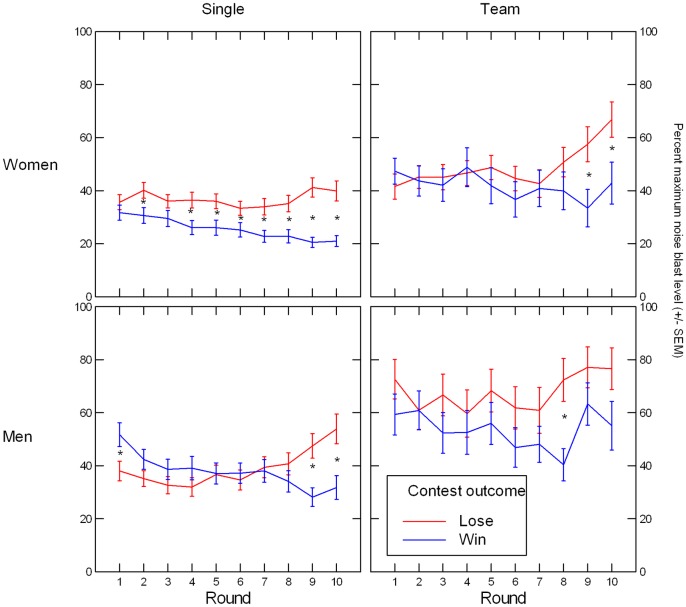
Illustration of Outcome x Gender x round effect, which was significant in single-competition participants, but not in team-competition participants (here, only the Outcome x round effect was significant). Asterisks denote significant (p < .05) differences between winners and losers on a given round.

Because noise blast level settings depended on individual decisions in the single condition, but on dyadic decisions in the team condition, we ran two separate regression models for the levels of the factor Condition, with Contest Outcome and Sex as between-subject factors and Round as within-subjects factor. In the single condition, we obtained a significant main effect of sex, *F*(1, 164) = 6.89, *p* = .009, *η*^*2*^ = .040, with women (*n* = 81, *M* = 31.84, *SD* = 16.63) showing lower overall aggression than men (*n* = 86, *M* = 38.43, *SD* = 20.23). We also obtained a significant three-way interaction of the experimental factors, *F*(9, 1467) = 2.68, Greenhouse-Geisser (G-G) *p* = .014, *η*^*2*^ = .016 (see Fig 8). Follow-up analyses indicated that the interaction was based on significant Outcome x Round interactions among women, *F*(9, 711) = 7.48, G-G *p* = .00001, *η*^*2*^ = .087 (linear trend: *F*(1, 79) = 13.63, *p* = .0004), and men, *F*(9, 756) = 9.88, G-G *p* < .0000005, *η*^*2*^ = .105 (linear trend: *F*(1, 84) = 32.11, *p* < .0000005). As depicted in Fig 8, these effects were in turn due to decreasing aggression among winners (women: *F*(9, 378) = 12.78, G-G *p* < .0000005, *η*^*2*^ = .233; men: *F*(9, 387) = 4.94, G-G *p* = .00005, *η*^*2*^ = .103) and increasing aggression among losers (women: *F*(9, 333) = 2.97, G-G *p* = .022, *η*^*2*^ = .074; men: *F*(9, 369) = 8.59, G-G *p* < .000005, *η*^*2*^ = .173). The root of the three-way interaction, however, lay in the much steeper aggression slope in male compared to female losers, *F*(9, 702) = 4.12, G-G *p* = .002, *η*^*2*^ = .050—a slope difference that did not emerge for male and female winners, *F*(9, 765) = 1.24, G-G *p* = .28, *η*^*2*^ = .014.

For the team condition, there was a significant gender main effect, *F*(1, 76) = 8.83, *p* = .004, *η*^*2*^ = .104, reflecting lower overall aggression in female teams (*n* = 40, *M* = 45.33, *SD* = 22.13) compared to male teams (*n* = 39, *M* = 59.95, *SD* = 24.19), and a Round x Outcome interaction, *F*(9, 684) = 2.88, G-G *p* = .006, *η*^*2*^ = .036 (linear trend: *F*(1, 76) = 13.90, *p* = .0004). The latter effect was due mainly to losing teams showing increasing aggression across the contest, *F*(9, 342) = 4.51, G-G *p* = .0002, *η*^*2*^ = .106, whereas winning teams’ aggression did not systematically change, *F*(9, 351) = 1.60, G-G *p* = .15, *η*^*2*^ = .039 (see Fig 8).

When we added nPower and nAffiliation to analyses in the single condition, we found no main or interaction effects of motive variables with other experimental factors, *p*s > .05. Corresponding analyses for the team condition, which featured two variables for each motive (one for each partner in a given team), only revealed an Outcome x nPower_1_ x nPower_2_ effect, *F*(1, 70) = 4.77, *p* = .03, *η*^*2*^ = .064. While the power motive of members of winning teams had no significant effect on aggression levels, there was a nPower_1_ x nPower_2_ effect among losing teams, *B* = -11.46, *SE* = 4.71, *t*(35) = -2.43, *p* = .02. Interaction plots (see supplement) indicated that in losing teams average aggression was higher if only one team member was high in in nPower than if both teammates were either high or low in nPower.

### Associations of aggression with hormones and motives

We explored whether baseline hormone levels, residualized hormone changes (averaged across T2 and T3), and motive measures were systematically associated with aggression (average and linear slope across 10 rounds). For an overview, see Tables 2 (single) and 3 (team). The most clear-cut hormone-aggression relationship emerged for C. In the single condition, a simultaneous regression of average aggression on C at T1, residualized changes in C, sex, and the interaction terms of gender and hormone variables revealed effects for Sex x C (T1), *B* = -31.08, *SE* = 9.33, *t*(134) = -3.33, *p* = .001, and Sex x C changes, *B* = -32.73, *SE* = 18.12, *t*(134) = -1.81, *p* = .073. Follow-up regressions broken down by sex revealed negative associations between aggression and baseline C in men, *B* = -16.09, *SE* = 6.14, *t*(70) = -2.62, *p* = .011, and C changes, *B* = -29.72, *SE* = 13.13, *t*(70) = -2.26, *p* = .027. In women, only the effect of baseline C on aggression became significant, *B* = 14.99, *SE* = 6.75, *t*(64) = 2.22, *p* = .03, indicating a *positive* association between C and aggression. When we simultaneously regressed aggression (average) on teams' averaged C at T1 and averaged C changes in the team condition, both hormone variables were negatively associated with aggression; for C at T1: *B* = -28.72, *SE* = 11.26, *t*(58) = -2.55, *p* = .013; for C changes: *B* = -56.92, *SE* = 25.09, *t*(58) = -2.27, *p* = .027. These effects were not moderated by sex in the team condition. We found no other systematic associations between aggression, averaged or as slope, and motive or hormone measures. We also specifically tested, separately for individuals and teams, for interactive effects between T baseline (or changes) and C baseline (or change) on averaged aggression and aggression slope to evaluate a dual-hormone hypothesis of aggression [62], but without significant results for winners or losers, women or men. Although Tables 2 and 3 include a few significant, local correlations, we refrain from interpreting them due to the large number of correlations tested.

**Table 2:** Pearson correlations between aggression (average, linear slope across 10 rounds), motives, and hormone baselines and changes in the single-competition condition.

	Men			NC women			OC women		
	Average	Slope	*n*	Average	Slope	*n*	Average	Slope	*n*
Win									
nPower	-0.07	0.03	44	-0.10	0.14	17	0.09	**-0.39**	26
nAffiliation	-0.15	0.10	44	-0.09	-0.21	17	-0.16	0.20	26
Testosterone T1	**-**0.29	-0.25	42	0.26	0.17	15	0.47	-0.13	21
Testosterone change	-0.08	-0.10	42	-0.03	0.07	15	-0.25	0.32	21
Estradiol T1	-0.22	0.15	32	-0.01	-0.10	15	0.01	0.01	25
Estradiol change	-0.10	-0.07	32	0.00	0.42	15	0.15	-0.20	25
Progesterone T1	-0.05	-0.30	40	0.33	0.08	14	-0.04	-0.24	25
Progesterone change	-0.05	-0.02	40	-0.47	0.15	14	-0.19	0.08	25
Cortisol T1	**-0.48**	-0.02	36	0.54	-0.22	10	0.40	**-0.49**	23
Cortisol change	**-0.34**	0.24	36	0.27	-0.03	10	0.06	0.31	23
Lose									
nPower	0.22	0.27	42	0.14	-0.06	16	-0.18	-0.23	22
nAffiliation	-0.03	-0.27	42	-0.08	0.42	16	0.37	0.03	22
Testosterone T1	0.07	-0.03	41	-0.15	0.19	16	0.11	-0.15	21
Testosterone change	0.11	0.11	41	0.12	0.20	15	0.09	0.15	21
Estradiol T1	-0.12	0.15	33	-0.43	0.03	16	0.16	-0.23	24
Estradiol change	-0.11	0.05	33	0.07	0.05	16	0.11	-0.27	24
Progesterone T1	-0.18	0.12	34	-0.22	0.01	15	0.32	-0.21	20
Progesterone change	0.07	0.13	34	0.29	0.28	15	0.32	0.05	17
Cortisol T1	-0.16	-0.11	37	0.31	0.04	15	-0.03	0.07	20
Cortisol change	-0.14	0.09	37	-0.09	-0.03	15	-0.02	0.09	20

Bold correlations: *p* < .05.

Hormone levels at T1 represent transformed values and hormone changes represent residualized change scores derived in the manner described in the text. *n* = individual participants.

**Table 3:** Pearson correlations between aggression (average, linear slope across 10 rounds), motives (team average), and hormone baselines and changes (team averages) in the team-competition condition.

	Men			Women		
	Average	Slope	*n*	Average	Slope	*n*
Win						
nPower	0.03	-0.05	19	-0.19	-0.04	20
nAffiliation	-0.10	0.27	19	0.27	-0.05	20
Testosterone T1	-0.16	0.02	19	0.13	0.07	20
Testosterone change	0.01	-0.02	19	-0.07	0.17	19
Estradiol T1	-0.32	-0.11	16	0.21	0.40	20
Estradiol change	-0.13	-0.31	16	-0.02	-0.25	20
Progesterone T1	-0.27	-0.27	19	0.08	0.00	20
Progesterone change	-0.26	-0.06	19	0.30	0.22	20
Cortisol T1	-0.27	-0.37	19	-0.22	-0.09	17
Cortisol change	-0.23	0.20	19	-0.30	0.03	17
Lose						
nPower	-0.27	-0.03	20	0.37	0.38	20
nAffiliation	-0.19	**-0.49**	20	0.03	-0.05	20
Testosterone T1	0.13	-0.10	16	0.27	0.12	17
Testosterone change	0.35	-0.26	16	-0.03	-0.15	17
Estradiol T1	-0.27	-0.48	4	-0.24	-0.03	19
Estradiol change	**0.97**	-0.34	4	0.12	0.15	19
Progesterone T1	-0.24	-0.34	16	0.07	0.04	18
Progesterone change	0.27	0.40	16	-0.29	-0.39	18
Cortisol T1	-0.45	0.35	14	-0.43	0.21	12
Cortisol change	-0.21	0.18	14	0.21	0.02	12

Bold correlations: *p* < .05.

Hormone levels at T1 represent transformed values and hormone changes represent residualized change scores derived in the manner described in the text. *n* = teams.

## Discussion

Our aim in the present study was to test, within one laboratory competition paradigm, men's and women's responses to a performance-based contest and its outcome, both for one-on-one and team competitions, in terms of hormonal changes relevant for dominance and affiliation as well as in terms of aggression towards the opponent(s). We verified the effectiveness of our contest paradigm by examining participants' affective responses to the contest outcome and their suspicions. Across all conditions resulting from the 2 x 2 factorial of sex and single/team condition, winners were significantly happier after the contest than losers, and the overall size of this effect was in the medium range. Moreover, at the end of the data collection session, less than 10% of our sample voiced any kind of suspicion about the veridicality of the contest outcome or whether they actually competed against someone else. Importantly, we found that even those participants who later voiced suspicions had virtually the same affective response to the contest outcome as participants who remained unsuspecting until the end. We interpret these findings as supporting the methodological validity of our laboratory competition paradigm. We are therefore inclined to put trust in our hormonal and behavioral results, which we summarize as follows.

### Testosterone: Winner effect for women competing as teams and for men competing individually

For T, we failed to observe a general effect of higher post-contest values in winners than in losers. Instead, we found that men who won as individuals strongly differed from men who won as teams–the former showed a T increase, the latter a T decrease. The opposite pattern emerged for NC women: individual winners showed a T decrease, whereas winning teams showed a T increase. Except for winning female teams, who also differed from losing female teams and their decreasing T levels, we obtained little evidence for significant differences between winners and losers. These findings suggest that, as suggested in the introduction, the social context is more critical for how women and men respond to winning a contest than the comparison with how they respond to losing. If taken at face value, our findings suggest that the winner/loser effect, defined now more narrowly as a T increase in response to a dominance success (see also [14]), can be found in men when they successfully compete against other men as individuals, but not when they successfully compete against other men as teams. In contrast, in NC women the winner/loser effect can be observed in winning teams, but not in winning individuals. Our findings thus indicate that whether researchers will observe a winner/loser effect in humans critically depends both on gender and on the social context of the competition.

### nPower-dependent testosterone responses: Replication of Schultheiss et al [34] effect for women, paradox results for men

In line with our hypotheses, our findings also show that contest outcome interacts with nPower in shaping T responses, albeit in different ways for women and men. Regardless of whether participants competed as individuals or teams, in NC women who won the contest and in men who lost the contest, higher nPower predicted a greater increase in post-contest T. The former finding replicates the observation by Schultheiss et al al [34] that in women nPower predicts higher T post-contest. The latter finding, however, is the opposite of what these authors observed for male losers and requires explanation. We think that a major reason for the differences between our and their findings is the inclusion of an explicit aggression component in the present study that was absent in the earlier study. In the Schultheiss et al [34] study, losers only learned that on most rounds they had not been able to keep up with their opponent. In our study, losers additionally received their opponents' aggression in the form of a noise blast. We suggest that this represented a type of provocation that reverberated with male losers' nPower, resulting in sympathetic activation and ultimately, as a consequence of adrenaline's stimulating effect on the testes (see [38, 63]), in increasing T. For women, such a stimulating adrenal effect on the ovaries has not been documented so far, which may explain why female losers did not show the nPower-dependent T increase we observed in male losers. Further research is needed to substantiate the validity of this interpretation of our results.

### Estradiol: Replication of nPower x Outcome effect reported by Stanton and Schultheiss [29]

For E, we found scant evidence for direct main or interaction effects of experimentally varied situational variables and gender, except for a trend for NC women in the individual-competition to register a post-contest E increase that was not observed in any other participant group. This finding may reflect a similar phenomenon as the warm-up effect for E observed in women preparing for competitive sports event [14]. However, findings became more differentiated once variations in nPower were taken into account, which interacted with contest outcome to predict NC women's (but no other group's) E changes. Replicating an interaction effect reported by Stanton and Schultheiss [29], higher nPower predicted a greater drop in E after losing, but not after winning a contest. In keeping with the notion of E as a female dominance hormone [29, 39], we speculate that such a drop in E may have an adaptive function by lowering the likelihood that an individual will acquire and maintain behaviors that lead to a defeat ([64, 65]; see [34], for related arguments and findings concerning the modulatory role of T in the acquisition of behaviors instrumental for successful dominance).

### Progesterone: Evidence for association with nAffiliation in women, depending on team versus individual competition

Variations in the situation and participants' gender yielded no direct or interacting effect on post-contest P, except for the observation of higher post-contest P in NC women compared to OC women or men. However, as expected based on previous observations of a specific association of P with nAffiliation [41], this motivational need predicted a post-contest P increase from immediately after the end of the competition until 20 min later in women who had been in a team and a mirror-image decrease in women who had competed alone. Neither contest outcome nor, in women, OC use moderated this finding. Our finding of a positive association between nAffiliation and P increases in a team context is broadly consistent with the notion that P is linked to affiliative motivation and behavior, provided that there is a situational opportunity to affiliate with another person [6]. This was the case in the team-competition condition, but not in the individual-competition condition. It is currently unclear why we failed to see a similar effect for men; despite inconsistencies in the observed associations between nAffiliation and P in men ([40, 41]), the published literature on the link between nAffiliation and P so far does not provide a compelling explanation for such a differential effect. Because ours was the first study to look at the nAffiliation-P link in the context of a stressful interpersonal challenge, we speculate that this context helped to elicit a gendered response to dealing with such a stressor: tend-and-befriend in women, fight-or-flight in men [43]. The former strategy is of course much more likely than the latter to be associated with affiliative motivation and its hormonal correlates. As Wirth [6] has pointed out, in a tend-and-befriend context, P's role may be anxiolytic and down-regulate stress axis activation.

### Effects of oral contraceptive use in women point to gonadal source testosterone and estradiol effects and adrenal source for progesterone effects

One notable aspect of our findings for the gonadal steroids T, E and P is the OC effect on women's hormonal responses and its implications for the origin of those responses. For T and E, we observed an absence of contest outcome effects–be they due purely to experimentally manipulated factors or be they due to interactions with nPower–in OC women and substantial effects in NC women. In contrast, OC use appeared to make no difference for the interactive effects of nAffiliation and competition type for women's P changes. Because OC regulate the release of sex steroids from the gonads but not from the adrenals, our results suggest that effects for T and E were mediated by hormone release from the gonads, but not the adrenals, and effects for P were mediated by hormone release from the adrenals, but not the gonads. Gonadal function was suppressed in OC women only, but not pharmacologically altered in any of the men we tested. Although our interpretation therefore currently holds for women specifically, we see no compelling reason to assume a completely different scenario for men [38]. We also emphasize that our interpretation specifically concerns contest *outcome* effects, because participation in a competition can elicit overall changes in T that appear to be unaffected by OC use and hence may be of adrenal origin [66].

### Cortisol: Female teams show post-win increase; nPower x Outcome effect in male teams represents partial replication of Wirth et al [37]

For C, we observed outcome-based effects only in the team competition. Here, the effect was driven by women, with winners showing higher post-contest C than losers. In the one-on-one condition, only participant gender affected post-contest C, with women showing greater increases than men. Again, these effects of situational factors and participant gender turned out to be moderated by nPower. In male teams, we observed the predicted association between nPower and C increases after losing, but not after winning the contest. These findings contrasted with observations in the individual-competition condition, where regardless of contest outcome nPower generally predicted lower post-contest C in women only.

The findings involving nPower are only in partial agreement with those reported by Wirth et al [37], who observed, for women and men alike, an association between higher nPower and post-contest C increases in losers, but not in winners. Because their study focused on competitions between individuals, the lack of agreement with our one-on-one condition is particularly noteworthy. Why did we not see the same positive association in male and female one-on-one contest losers? One reason may simply be test power. Perhaps the Wirth et al [37] overestimated the effect size of their findings or our sample size in the individual-contest condition was not large enough to reliably detect the effect or a combination of both. But continuing our earlier argument, we think it is more likely that the psychological situation in the present study was a different one for individually competing participants compared to the Wirth et al [37] study because of the addition of the aggression task. The option of setting noise blasts for the opponent may have helped to restore a sense of control in power-motivated losers, thus deactivating the hypothalamic-pituitary-adrenal axis, a system that is normally engaged by uncontrollable stressors [67]. By the same token, the situation changed for participants in the winning condition: in addition to beating their opponents, in the present study they could apply additional noise-blast punishment, but also risked retaliation during the contest. The mindsets of losers and winners are readily apparent in Fig 8, with escalating levels of aggression in losers and attenuated aggression in winners. To test whether these explanations have merit, it would be fruitful to examine the role of aggression in future studies that adopt the same competition paradigm we describe here, but vary the presence or absence of the aggression task.

### Aggression: Losers, men, and teams have higher (rising) aggression levels than winners, women, or individuals; little evidence for role of motives

Our findings concerning aggression can be summarized as follows. Besides replicating the gender difference favoring men observed in other studies [68, 69], we found that losers overall, and men in the one-on-one competition in particular, increased their aggression levels throughout the contest, moving from a situation in which the final outcome was not apparent (i.e., first half) to one in which it became increasingly clear that they were defeated and in which they also repeatedly received noise blasts after each lost round. The latter aspect in particular can be interpreted as a form of provocation, which is known to increase individuals' aggression levels [69]. Although not directly tested due to the asymmetric nature of the aggression data (individual data in the one-on-one condition, dyadic data in the team condition due to teams jointly setting noise blast levels), at the descriptive level we also observed overall higher aggression from teams than from individuals, a finding that is consistent with the frequently observed amplification of aggression in primate and human groups [70].

Notably, apart from a weak effect suggesting that in losing teams, aggression was higher if one team member only was high in nPower (as compared to both or neither), we found little evidence for associations between participants' implicit motives, either by themselves or in interaction with gender and experimental factors, and aggression. This dearth of findings makes sense if one considers that after (early) childhood [45], blatantly aggressive behavior is not usually instrumental for having lasting impact on others, the main incentive for nPower. In addition, such behavior incurs the risk of retaliation and thus becoming the object of others' attempt at influence, a clear disincentive for power-motivated individuals. Viewed from this perspective, the aggression component included in the present study may represent a largely discarded behavioral strategy for individuals seeking to have impact on others and, due to earlier learning experiences, may not be viewed and used as a viable means of exerting influence. This interpretation is also consistent with accounts that clearly separate aggression and dominance in primates ([71–73]) and that suggest that in humans, aggression is primarily defensive, not dominance-seeking [74]. Likewise, nAffiliation is an unlikely candidate for the attenuation of aggression in the competition context we studied. Although affiliation-motivated individuals are generally interested in maintaining friendly and thus non-aggressive relationships with others, this is more true for individuals who they perceive to be similar to them and not for individuals who they view as different from them (e.g., the opponents in the present study; see [75]). The latter case is apt to neutralize whatever aggression-attenuating effect nAffiliation may have. We therefore interpret our finding of increasing aggression in contest losers as supportive of Albert et al's [74] hypothesis that human aggression is primarily defensively oriented and its close relative, the frustration-aggression hypothesis. According to the latter theory in its present form [76], unexpected frustration of one's goals (such as failing to win most rounds in the losing condition of the present study) as well as aversive stimulation (such as receiving repeated noise blasts as a consequence of not winning most rounds) lead to negative affective states, which in turn increase the likelihood of aggressing against others.

### Aggression: Association with baseline cortisol and cortisol changes, but not other hormones

Our exploratory analyses of associations between implicit motives and hormones on the one hand and aggression levels on the other revealed no systematic associations of initial or changing hormones related to male (T) or female (E) dominance with aggression means and slopes. This indicates that neither individual differences in these hormones nor contest-induced changes accounted for variations in aggression and reinforces our argument that in humans, motivational systems supporting dominance cannot be equated with systems that are responsible for aggressive behavior. This conclusion is also consistent with reviews of the T-aggression link that stress the inconsistency of this link, particularly in humans [74, 77]. (Note, however, that some research suggests that effects of contest-induced T increases on aggression may only become apparent on subsequent tasks that provide an outlet for aggression; see [78]). Neither did we obtain associations between initial or changing P and aggression, suggesting that P is neither an inhibiting nor a facilitating hormonal factor in aggression.

Instead, we obtained robust evidence for a role of C, both at baseline and as contest-related changes, in individuals' average aggression levels. In general, these associations were negative, suggesting that low or declining C predicted increased aggression, a finding that strongly corroborates other reports of a negative relationship between C and aggression ([79, 80]). The only exception from this pattern was that in women competing individually, higher C at baseline predicted higher aggression during the contest. We suggest that the key for understanding these apparently contradictory results may be the distinction between fear-related and impulsive (i.e., frustration-based) aggression. Reviewing the pertinent work in humans, Kalin [81] argues that fear-related aggression is marked by high C, whereas impulsive aggression is marked by low C. According to this interpretation, women with high baseline C participating in one-on-one competitions aggressed out of a sense of fear, perhaps because they found the situation of having to compete against another woman and being exposed to her aggression as particularly threatening. This interpretation also finds support in our observation that women in this condition showed a general post-contest C increase, regardless of winning or losing. In all other participants in all other conditions, in contrast, low C was associated with a disinhibition of aggressive impulses, perhaps due to the exposure to unsuccessful contest rounds and the associated experience of another's noise blasts.

### Limitations and future directions

Our study has clear strengths, such as a design integrating many aspects of competition usually studied separately, a large sample, the simultaneous assessment of four hormonal parameters, the high standardization of the computer-based competition paradigm, independent checks of its methodological validity, and the procedural assessment of implicit motivational needs relevant for predicting hormonal and behavioral responses to competitions. But it is not without limitations. Some resided in our assessment of implicit motives. Our picture-story measure of motives was geared towards the differentiated assessment of nPower, but was less suitable in terms of score distributions for the assessment of nAffiliation, which may explain the relative lack of effects obtained for the latter motive. Researchers interested in a more detailed study of the contribution of nAffiliation to hormonal and behavioral responses to competitions, particularly when looking at teams, should therefore compile a picture story measure geared specifically towards the assessment of this motive [82]. Moreover, due to limitations in how much work each coder could do, we had four coders code the picture stories of four subsets of participants and later converted the scores obtained in each subset to z scores. Although this eliminated coder bias, it may also have obscured whatever valid differences in the means and variances there was between the subsets and thus imposed restrictions on whatever motive effects we were able to detect in our data set.

Other limitations involved our salivary hormone assays. Because not all participants provided sufficient amounts of sample for each sample, we were unable to assess all four hormones three times in all participants. This led to somewhat lower *N*s and hence lower test power for analyses involving hormones. In addition, like in previous studies [29], assessment of salivary E approached the boundaries of RIA-based detection of steroids, as reflected in high intrassay CVs. Despite this, we found that when the retest reliability across duplicates of samples is estimated via a correlation instead of an average CV, the resulting coefficient is well above .70, which can be considered satisfactory (see S1 File). Another limitation we encountered was the low recovery coefficient of our P assay, which may have restricted the validity of the variance in P measurements.

Some limitations of the present research pertain to the assessment of aggression. One such limitation resulted from the fact that in the one-on-one condition, participants set noise-blast levels individually, whereas in the two-on-two condition, they were set jointly by each team and thus reflected the aggression level of two participants, not one. This restricted our option of subjecting aggression levels from both conditions to one data-analytic design. We suggest that this problem can be circumvented in future studies by (a) having each member of a team enter a noise-blast level and informing teams that a joint noise blast will be used based on the average of both entries and/or (b) using multilevel modeling for joint analyses of the data. Another limitation is due to interdependency of total received aggression with total number of rounds lost: It is difficult to tease apart whether losers aggressed more because they received more aggression or because they lost more rounds than winners. One way to deal with this dilemma in future studies would be to omit the application or reception of aggression during the contest itself and instead to tell participants that noise blast opportunities can be “earned” across rounds and can be meted out to the opponent(s) once the contest is over (and vice versa for the opponent).

Yet another limitation may be the nature of our competition task. Previous research shows that real-world competitions with higher stakes, such as athletic competitions, can result in more clear-cut hormonal differences after victory and defeat than laboratory contests like the one we used [14, 83]. However, it is currently unclear to what extent this difference in effect sizes is due to a self-selection effect of people participating in real-world contests and to what extent it represents a confound between hormonal responses to winning and losing and uncontrolled factors that determine outcomes in real-world contests.

Finally, given the scope of our study in terms of conditions realized and variables measured and the number of statistical tests involved, we cannot rule out the possibility that some findings are false positives. But neither can we rule out the possibility that some of the expected effects we failed to observe may represent false negatives. Thus, the findings we report here need to be replicated, a task we have tried to facilitate by programming all tasks and instructions on the computer and thus making all aspects of running this study available for sharing with other labs.

### Conclusion

To our knowledge, the present research is the first to study in the laboratory the endocrine and behavioral responses of men and women to individual and team competitions with experimentally controlled outcomes in one experimental and analytical design. Our findings support the usefulness of such an integrated approach by uncovering nuances in participants' responses to winning and losing, depending on gender and competition type. More specifically, they suggest that factors related to gender or the competition situation alone may often not be sufficient for gaining a deeper understanding of individuals' hormonal and behavioral responses to competition; rather, the confluence of these factors with individuals' dispositional motivational needs, and hence what they deem rewarding or punishing, needs to be taken into account. They also suggest that even in the context of a dominance contest, aggressive behavior depends less on motivational and hormonal factors directly linked to dominance and more on stress and its endocrine basis.

## Supporting information

S1 Dataset Oxford et al data set on which all reported analyses are based. (CSV)

S1 Analysis SYSTAT 13 syntax for generating the analyses, tables and graphs reported in this paper. It can be viewed in any text editor.
(SYC)

S1 File Supporting information regarding reliability of the salivary estradiol assays, inter-hormone correlations, and effects of team members’ motives on punishment level. (DOCX)
