# Supplementary material for: Endocrine and aggressive responses to competition are moderated by contest outcome, gender, individual versus team competition, and implicit motives
Source: PLoS One. 2017 Jul 27;12(7):e0181610. doi: 10.1371/journal.pone.0181610 (PMC5531467; doi:10.1371/journal.pone.0181610)
Supplement: S1 File — (DOCX) [file pone.0181610.s003.docx]

**S3 Supporting Information**

**Supplement to Oxford et al „Endocrine and aggressive responses to competition are moderated by contest outcome, gender, individual versus team competition, and implicit motives”, *PLOS ONE***

1. Reliability of estradiol radioimmunoassay data

In light of the high intra-assay CV% values of our estradiol assay, we examined the reliability of the salivary estradiol measurements from the perspective of correlation analysis: How well do duplicate measurements of the same samples correlate with each other? For the 672 saliva samples for which we had sufficient material to assay estradiol in duplicate, we obtained a Pearson correlation of *r* = .861 (see Figure 1). Because the estradiol concentration data were skewed, we also subjected all measurements to a log transformation after adding a constant of 1 (i.e., log E = log[1 + E]). The Pearson correlation for duplicate measurements transformed in this manner was *r* = .792 (see Figure 2). Corresponding Spearman rank-order correlations were *r* = .719 for both untransformed and transformed estradiol measurements. Thus, from a classical test theory perspective, the reliability coefficients we obtained for our estradiol assay suggest that 79% (Pearson) or 72% (Spearman) of the observed variability in estradiol levels within one sample represent true differences in estradiol levels; the rest is measurement error. We interpret this finding to mean that despite the problems associated with assaying a steroid hormone as rare in its unbound form as estradiol, we still get an estimate of a participant's salivary estradiol with satisfactory precision (see Martin & Bateson, 1993, p. 119). Results for the retest reliability for all three saliva samples collected (see next section) support this conclusion: retest reliability for estradiol does not appear to be substantially lower than retest reliability coefficients for other salivary hormone measurements.

Figure 1 Scatterplot with regression line for untransformed estradiol duplicate measurements A and B

Figure 2 Scatterplot with regression line for log-transformed estradiol duplicate measurements A and B

1. Correlations of hormone measurements at T1 through T3 and hormone changes

In the following, we report Pearson correlations for all four hormones assessed. For each hormone, we include measurements at T1 (baseline), T2 (immediately after the contest), and T3 (20 min after the contest) as well as residualized change scores based on averaged T2 and T3 values, with baseline values (T1) regressed from this average. Prior to averaging, residualization, and correlation analysis, all hormones were transformed as described in the paper to correct for distribution skew and to lessen the effects of outliers. For testosterone (T), estradiol (E), and progesterone (P) – that is, for the three hormones that are affected by the use of oral contraceptives in women – we created residualized change scores (abbreviated as *ch*) separately for naturally cycling (NC) women, oral-contraceptive-using (OC) women, and men (i.e., hormone levels averaged across T2 and T3 were regressed on hormone levels at T1 within each group and residualized change scores were calculated based on the regression equations obtained for each group). Because oral contraceptive use and gender did not have main effects on baseline salivary cortisol (C), residualized change scores for averaged post-contest measurements were calculated based on the entire sample.

**NC women**

| **Pearson Correlation Matrix** | | | | | | | | | | | | | | | | |
| --- | --- | --- | --- | --- | --- | --- | --- | --- | --- | --- | --- | --- | --- | --- | --- | --- |
|  | **T T1** | **T T2** | **T T3** | **T ch** | **E T1** | **E T2** | **E T3** | **E ch** | **P T1** | **P T2** | **P T3** | **P ch** | **C T1** | **C T2** | **C T3** | **C ch** |
| T T1 | 1.00 |  |  |  |  |  |  |  |  |  |  |  |  |  |  |  |
| T T2 | 0.88 | 1.00 |  |  |  |  |  |  |  |  |  |  |  |  |  |  |
| T T3 | 0.86 | 0.92 | 1.00 |  |  |  |  |  |  |  |  |  |  |  |  |  |
| T ch | 0.00 | 0.43 | 0.45 | 1.00 |  |  |  |  |  |  |  |  |  |  |  |  |
| E T1 | 0.19 | -0.01 | -0.01 | -0.28 | 1.00 |  |  |  |  |  |  |  |  |  |  |  |
| E T2 | 0.19 | 0.05 | 0.05 | -0.12 | 0.86 | 1.00 |  |  |  |  |  |  |  |  |  |  |
| E T3 | 0.13 | -0.04 | -0.03 | -0.18 | 0.82 | 0.90 | 1.00 |  |  |  |  |  |  |  |  |  |
| E ch | 0.01 | 0.04 | 0.04 | 0.18 | 0.00 | 0.47 | 0.52 | 1.00 |  |  |  |  |  |  |  |  |
| P T1 | 0.20 | -0.08 | -0.01 | -0.49 | 0.61 | 0.47 | 0.56 | -0.02 | 1.00 |  |  |  |  |  |  |  |
| P T2 | 0.17 | -0.08 | -0.01 | -0.44 | 0.50 | 0.42 | 0.54 | 0.11 | 0.94 | 1.00 |  |  |  |  |  |  |
| P T3 | 0.11 | -0.07 | 0.00 | -0.33 | 0.49 | 0.44 | 0.58 | 0.19 | 0.92 | 0.97 | 1.00 |  |  |  |  |  |
| P ch | -0.07 | -0.01 | 0.02 | 0.15 | -0.02 | 0.13 | 0.22 | 0.38 | 0.00 | 0.32 | 0.43 | 1.00 |  |  |  |  |
| C T1 | 0.18 | 0.04 | -0.02 | -0.31 | 0.33 | 0.21 | 0.18 | -0.16 | 0.13 | 0.12 | 0.08 | -0.05 | 1.00 |  |  |  |
| C T2 | 0.19 | 0.07 | 0.02 | -0.26 | 0.31 | 0.25 | 0.30 | 0.03 | 0.19 | 0.14 | 0.13 | -0.10 | 0.82 | 1.00 |  |  |
| C T3 | 0.16 | 0.05 | 0.09 | -0.16 | 0.26 | 0.22 | 0.30 | 0.09 | 0.17 | 0.14 | 0.13 | -0.06 | 0.61 | 0.88 | 1.00 |  |
| C ch | 0.07 | 0.04 | 0.11 | 0.03 | 0.06 | 0.12 | 0.25 | 0.27 | 0.13 | 0.08 | 0.11 | -0.07 | -0.04 | 0.51 | 0.73 | 1.00 |

| **Pairwise Frequency Table** | | | | | | | | | | | | | | | | |
| --- | --- | --- | --- | --- | --- | --- | --- | --- | --- | --- | --- | --- | --- | --- | --- | --- |
|  | **T T1** | **T T2** | **T T3** | **T ch** | **E T1** | **E T2** | **E T3** | **E ch** | **P T1** | **P T2** | **P T3** | **P ch** | **C T1** | **C T2** | **C T3** | **C ch** |
| T T1 | 53 |  |  |  |  |  |  |  |  |  |  |  |  |  |  |  |
| T T2 | 51 | 54 |  |  |  |  |  |  |  |  |  |  |  |  |  |  |
| T T3 | 49 | 52 | 53 |  |  |  |  |  |  |  |  |  |  |  |  |  |
| T ch | 51 | 51 | 49 | 51 |  |  |  |  |  |  |  |  |  |  |  |  |
| E T1 | 50 | 52 | 51 | 49 | 59 |  |  |  |  |  |  |  |  |  |  |  |
| E T2 | 50 | 52 | 51 | 49 | 59 | 59 |  |  |  |  |  |  |  |  |  |  |
| E T3 | 51 | 53 | 52 | 50 | 59 | 59 | 60 |  |  |  |  |  |  |  |  |  |
| E ch | 50 | 52 | 51 | 49 | 59 | 59 | 59 | 59 |  |  |  |  |  |  |  |  |
| P T1 | 49 | 50 | 48 | 48 | 52 | 52 | 53 | 52 | 57 |  |  |  |  |  |  |  |
| P T2 | 50 | 51 | 50 | 48 | 57 | 57 | 58 | 57 | 57 | 62 |  |  |  |  |  |  |
| P T3 | 49 | 50 | 50 | 47 | 53 | 53 | 54 | 53 | 54 | 58 | 58 |  |  |  |  |  |
| P ch | 49 | 50 | 48 | 48 | 52 | 52 | 53 | 52 | 57 | 57 | 54 | 57 |  |  |  |  |
| C T1 | 42 | 42 | 42 | 42 | 42 | 42 | 43 | 42 | 42 | 42 | 42 | 42 | 43 |  |  |  |
| C T2 | 42 | 42 | 42 | 42 | 42 | 42 | 43 | 42 | 42 | 42 | 42 | 42 | 43 | 43 |  |  |
| C T3 | 42 | 42 | 42 | 42 | 42 | 42 | 43 | 42 | 42 | 42 | 42 | 42 | 43 | 43 | 43 |  |
| C ch | 42 | 42 | 42 | 42 | 42 | 42 | 43 | 42 | 42 | 42 | 42 | 42 | 43 | 43 | 43 | 43 |

| **Matrix of Probabilities** | | | | | | | | | | | | | | | | |
| --- | --- | --- | --- | --- | --- | --- | --- | --- | --- | --- | --- | --- | --- | --- | --- | --- |
|  | **T T1** | **T T2** | **T T3** | **T ch** | **E T1** | **E T2** | **E T3** | **E ch** | **P T1** | **P T2** | **P T3** | **P ch** | **C T1** | **C T2** | **C T3** | **C ch** |
| T T1 | 0.00000 |  |  |  |  |  |  |  |  |  |  |  |  |  |  |  |
| T T2 | 0.00000 | 0.00000 |  |  |  |  |  |  |  |  |  |  |  |  |  |  |
| T T3 | 0.00000 | 0.00000 | 0.00000 |  |  |  |  |  |  |  |  |  |  |  |  |  |
| T ch | 1.00000 | 0.00188 | 0.00127 | 0.00000 |  |  |  |  |  |  |  |  |  |  |  |  |
| E T1 | 0.19039 | 0.92321 | 0.95346 | 0.05436 | 0.00000 |  |  |  |  |  |  |  |  |  |  |  |
| E T2 | 0.19113 | 0.73746 | 0.74998 | 0.39697 | 0.00000 | 0.00000 |  |  |  |  |  |  |  |  |  |  |
| E T3 | 0.37820 | 0.79947 | 0.83925 | 0.20922 | 0.00000 | 0.00000 | 0.00000 |  |  |  |  |  |  |  |  |  |
| E ch | 0.96739 | 0.75521 | 0.79100 | 0.22794 | 1.00000 | 0.00019 | 0.00003 | 0.00000 |  |  |  |  |  |  |  |  |
| P T1 | 0.16388 | 0.57731 | 0.95276 | 0.00041 | 0.00000 | 0.00045 | 0.00001 | 0.86508 | 0.00000 |  |  |  |  |  |  |  |
| P T2 | 0.22565 | 0.56555 | 0.93809 | 0.00183 | 0.00007 | 0.00116 | 0.00001 | 0.43431 | 0.00000 | 0.00000 |  |  |  |  |  |  |
| P T3 | 0.43413 | 0.65245 | 0.97837 | 0.02235 | 0.00020 | 0.00090 | 0.00000 | 0.16244 | 0.00000 | 0.00000 | 0.00000 |  |  |  |  |  |
| P ch | 0.61542 | 0.92913 | 0.89865 | 0.30527 | 0.88137 | 0.37242 | 0.11759 | 0.00491 | 1.00000 | 0.01424 | 0.00101 | 0.00000 |  |  |  |  |
| C T1 | 0.25997 | 0.79944 | 0.88772 | 0.04935 | 0.03402 | 0.18787 | 0.24920 | 0.32488 | 0.40191 | 0.44223 | 0.59502 | 0.74216 | 0.00000 |  |  |  |
| C T2 | 0.22858 | 0.67233 | 0.89492 | 0.09823 | 0.04325 | 0.10546 | 0.05055 | 0.83276 | 0.23722 | 0.36650 | 0.41397 | 0.54476 | 0.00000 | 0.00000 |  |  |
| C T3 | 0.30011 | 0.75451 | 0.55536 | 0.32169 | 0.10286 | 0.16566 | 0.05065 | 0.56133 | 0.28349 | 0.39398 | 0.41155 | 0.69529 | 0.00001 | 0.00000 | 0.00000 |  |
| C ch | 0.66339 | 0.78127 | 0.47455 | 0.85720 | 0.70847 | 0.43777 | 0.09996 | 0.07944 | 0.40870 | 0.60141 | 0.49341 | 0.67987 | 0.77658 | 0.00048 | 0.00000 | 0.00000 |

**OC women**

| **Pearson Correlation Matrix** | | | | | | | | | | | | | | | | |
| --- | --- | --- | --- | --- | --- | --- | --- | --- | --- | --- | --- | --- | --- | --- | --- | --- |
|  | **T T1** | **T T2** | **T T3** | **T ch** | **E T1** | **E T2** | **E T3** | **E ch** | **P T1** | **P T2** | **P T3** | **P ch** | **C T1** | **C T2** | **C T3** | **C ch** |
| T T1 | 1.00 |  |  |  |  |  |  |  |  |  |  |  |  |  |  |  |
| T T2 | 0.87 | 1.00 |  |  |  |  |  |  |  |  |  |  |  |  |  |  |
| T T3 | 0.81 | 0.89 | 1.00 |  |  |  |  |  |  |  |  |  |  |  |  |  |
| T ch | 0.00 | 0.45 | 0.53 | 1.00 |  |  |  |  |  |  |  |  |  |  |  |  |
| E T1 | 0.34 | 0.31 | 0.28 | 0.02 | 1.00 |  |  |  |  |  |  |  |  |  |  |  |
| E T2 | 0.26 | 0.24 | 0.18 | -0.03 | 0.79 | 1.00 |  |  |  |  |  |  |  |  |  |  |
| E T3 | 0.26 | 0.22 | 0.20 | 0.00 | 0.74 | 0.79 | 1.00 |  |  |  |  |  |  |  |  |  |
| E ch | 0.08 | 0.03 | 0.00 | -0.04 | 0.00 | 0.56 | 0.58 | 1.00 |  |  |  |  |  |  |  |  |
| P T1 | 0.25 | 0.21 | 0.19 | 0.02 | 0.20 | 0.32 | 0.23 | 0.24 | 1.00 |  |  |  |  |  |  |  |
| P T2 | 0.06 | 0.06 | 0.04 | 0.04 | 0.13 | 0.22 | 0.19 | 0.24 | 0.87 | 1.00 |  |  |  |  |  |  |
| P T3 | -0.06 | 0.00 | -0.02 | 0.17 | 0.10 | 0.25 | 0.22 | 0.31 | 0.77 | 0.88 | 1.00 |  |  |  |  |  |
| P ch | -0.33 | -0.16 | -0.16 | 0.28 | -0.13 | -0.06 | 0.01 | 0.21 | 0.00 | 0.43 | 0.57 | 1.00 |  |  |  |  |
| C T1 | 0.25 | 0.10 | 0.08 | -0.33 | 0.08 | 0.14 | 0.07 | 0.09 | 0.17 | 0.10 | 0.00 | -0.20 | 1.00 |  |  |  |
| C T2 | 0.12 | 0.02 | 0.02 | -0.22 | 0.07 | 0.13 | 0.06 | 0.07 | 0.07 | -0.01 | -0.10 | -0.21 | 0.89 | 1.00 |  |  |
| C T3 | 0.11 | 0.03 | 0.02 | -0.16 | 0.02 | 0.08 | 0.07 | 0.11 | 0.07 | 0.03 | -0.06 | -0.16 | 0.83 | 0.93 | 1.00 |  |
| C ch | -0.16 | -0.05 | -0.04 | 0.22 | -0.02 | -0.04 | 0.00 | -0.03 | -0.13 | -0.13 | -0.16 | -0.05 | 0.17 | 0.56 | 0.67 | 1.00 |

| **Pairwise Frequency Table** | | | | | | | | | | | | | | | | |
| --- | --- | --- | --- | --- | --- | --- | --- | --- | --- | --- | --- | --- | --- | --- | --- | --- |
|  | **T T1** | **T T2** | **T T3** | **T ch** | **E T1** | **E T2** | **E T3** | **E ch** | **P T1** | **P T2** | **P T3** | **P ch** | **C T1** | **C T2** | **C T3** | **C ch** |
| T T1 | 77 |  |  |  |  |  |  |  |  |  |  |  |  |  |  |  |
| T T2 | 73 | 79 |  |  |  |  |  |  |  |  |  |  |  |  |  |  |
| T T3 | 73 | 76 | 78 |  |  |  |  |  |  |  |  |  |  |  |  |  |
| T ch | 74 | 73 | 73 | 74 |  |  |  |  |  |  |  |  |  |  |  |  |
| E T1 | 73 | 76 | 75 | 71 | 90 |  |  |  |  |  |  |  |  |  |  |  |
| E T2 | 72 | 74 | 74 | 70 | 86 | 87 |  |  |  |  |  |  |  |  |  |  |
| E T3 | 71 | 75 | 73 | 70 | 84 | 82 | 86 |  |  |  |  |  |  |  |  |  |
| E ch | 72 | 75 | 74 | 70 | 89 | 86 | 84 | 89 |  |  |  |  |  |  |  |  |
| P T1 | 74 | 75 | 73 | 71 | 79 | 77 | 77 | 78 | 85 |  |  |  |  |  |  |  |
| P T2 | 71 | 73 | 71 | 68 | 79 | 76 | 76 | 78 | 81 | 85 |  |  |  |  |  |  |
| P T3 | 69 | 72 | 70 | 67 | 75 | 72 | 72 | 74 | 77 | 80 | 80 |  |  |  |  |  |
| P ch | 71 | 72 | 70 | 68 | 75 | 73 | 73 | 74 | 81 | 81 | 77 | 81 |  |  |  |  |
| C T1 | 62 | 64 | 63 | 62 | 62 | 60 | 61 | 61 | 61 | 58 | 57 | 58 | 64 |  |  |  |
| C T2 | 63 | 65 | 64 | 63 | 63 | 61 | 62 | 62 | 62 | 59 | 58 | 59 | 64 | 65 |  |  |
| C T3 | 63 | 65 | 64 | 63 | 63 | 61 | 62 | 62 | 62 | 59 | 58 | 59 | 64 | 65 | 65 |  |
| C ch | 62 | 64 | 63 | 62 | 62 | 60 | 61 | 61 | 61 | 58 | 57 | 58 | 64 | 64 | 64 | 64 |

| **Matrix of Probabilities** | | | | | | | | | | | | | | | | |
| --- | --- | --- | --- | --- | --- | --- | --- | --- | --- | --- | --- | --- | --- | --- | --- | --- |
|  | **T T1** | **T T2** | **T T3** | **T ch** | **E T1** | **E T2** | **E T3** | **E ch** | **P T1** | **P T2** | **P T3** | **P ch** | **C T1** | **C T2** | **C T3** | **C ch** |
| T T1 | 0.00000 |  |  |  |  |  |  |  |  |  |  |  |  |  |  |  |
| T T2 | 0.00000 | 0.00000 |  |  |  |  |  |  |  |  |  |  |  |  |  |  |
| T T3 | 0.00000 | 0.00000 | 0.00000 |  |  |  |  |  |  |  |  |  |  |  |  |  |
| T ch | 0.99999 | 0.00007 | 0.00000 | 0.00000 |  |  |  |  |  |  |  |  |  |  |  |  |
| E T1 | 0.00338 | 0.00564 | 0.01430 | 0.89125 | 0.00000 |  |  |  |  |  |  |  |  |  |  |  |
| E T2 | 0.02820 | 0.04051 | 0.11856 | 0.82183 | 0.00000 | 0.00000 |  |  |  |  |  |  |  |  |  |  |
| E T3 | 0.02563 | 0.05361 | 0.08274 | 0.98572 | 0.00000 | 0.00000 | 0.00000 |  |  |  |  |  |  |  |  |  |
| E ch | 0.50793 | 0.80113 | 0.98605 | 0.75007 | 1.00000 | 0.00000 | 0.00000 | 0.00000 |  |  |  |  |  |  |  |  |
| P T1 | 0.03032 | 0.06736 | 0.09873 | 0.87598 | 0.08218 | 0.00396 | 0.04034 | 0.03235 | 0.00000 |  |  |  |  |  |  |  |
| P T2 | 0.59728 | 0.59155 | 0.76311 | 0.72442 | 0.24633 | 0.05771 | 0.09331 | 0.03430 | 0.00000 | 0.00000 |  |  |  |  |  |  |
| P T3 | 0.65141 | 0.98091 | 0.90040 | 0.17306 | 0.38254 | 0.03797 | 0.06890 | 0.00681 | 0.00000 | 0.00000 | 0.00000 |  |  |  |  |  |
| P ch | 0.00546 | 0.17155 | 0.17285 | 0.02131 | 0.28044 | 0.62325 | 0.90328 | 0.07440 | 1.00000 | 0.00007 | 0.00000 | 0.00000 |  |  |  |  |
| C T1 | 0.04850 | 0.44423 | 0.54191 | 0.00955 | 0.53266 | 0.27380 | 0.60197 | 0.47037 | 0.17811 | 0.46242 | 0.98905 | 0.12593 | 0.00000 |  |  |  |
| C T2 | 0.34936 | 0.89909 | 0.89940 | 0.08636 | 0.60712 | 0.32746 | 0.65316 | 0.58205 | 0.61172 | 0.96975 | 0.45037 | 0.11874 | 0.00000 | 0.00000 |  |  |
| C T3 | 0.40527 | 0.80670 | 0.90358 | 0.21639 | 0.87143 | 0.52623 | 0.59252 | 0.40748 | 0.59629 | 0.84496 | 0.67617 | 0.21817 | 0.00000 | 0.00000 | 0.00000 |  |
| C ch | 0.20609 | 0.68684 | 0.73080 | 0.08141 | 0.84720 | 0.75755 | 0.99703 | 0.84496 | 0.30374 | 0.33320 | 0.22600 | 0.71545 | 0.17837 | 0.00000 | 0.00000 | 0.00000 |

**Men**

| **Pearson Correlation Matrix** | | | | | | | | | | | | | | | | |
| --- | --- | --- | --- | --- | --- | --- | --- | --- | --- | --- | --- | --- | --- | --- | --- | --- |
|  | **T T1** | **T T2** | **T T3** | **T ch** | **E T1** | **E T2** | **E T3** | **E ch** | **P T1** | **P T2** | **P T3** | **P ch** | **C T1** | **C T2** | **C T3** | **C ch** |
| T T1 | 1.00 |  |  |  |  |  |  |  |  |  |  |  |  |  |  |  |
| T T2 | 0.81 | 1.00 |  |  |  |  |  |  |  |  |  |  |  |  |  |  |
| T T3 | 0.82 | 0.86 | 1.00 |  |  |  |  |  |  |  |  |  |  |  |  |  |
| T ch | 0.00 | 0.52 | 0.51 | 1.00 |  |  |  |  |  |  |  |  |  |  |  |  |
| E T1 | 0.25 | 0.21 | 0.25 | 0.05 | 1.00 |  |  |  |  |  |  |  |  |  |  |  |
| E T2 | 0.18 | 0.22 | 0.20 | 0.12 | 0.69 | 1.00 |  |  |  |  |  |  |  |  |  |  |
| E T3 | 0.11 | 0.12 | 0.14 | 0.07 | 0.63 | 0.74 | 1.00 |  |  |  |  |  |  |  |  |  |
| E ch | -0.02 | 0.07 | 0.03 | 0.12 | 0.00 | 0.62 | 0.69 | 1.00 |  |  |  |  |  |  |  |  |
| P T1 | 0.21 | 0.15 | 0.17 | -0.03 | 0.44 | 0.45 | 0.36 | 0.16 | 1.00 |  |  |  |  |  |  |  |
| P T2 | 0.19 | 0.22 | 0.20 | 0.10 | 0.33 | 0.50 | 0.39 | 0.34 | 0.78 | 1.00 |  |  |  |  |  |  |
| P T3 | 0.16 | 0.24 | 0.20 | 0.16 | 0.29 | 0.45 | 0.44 | 0.39 | 0.59 | 0.72 | 1.00 |  |  |  |  |  |
| P ch | 0.05 | 0.20 | 0.13 | 0.24 | 0.01 | 0.27 | 0.28 | 0.44 | 0.00 | 0.52 | 0.72 | 1.00 |  |  |  |  |
| C T1 | 0.22 | 0.12 | 0.06 | -0.16 | 0.21 | 0.27 | 0.23 | 0.19 | 0.14 | 0.17 | 0.25 | 0.20 | 1.00 |  |  |  |
| C T2 | 0.16 | 0.19 | 0.06 | 0.00 | 0.25 | 0.38 | 0.29 | 0.31 | 0.22 | 0.36 | 0.37 | 0.36 | 0.82 | 1.00 |  |  |
| C T3 | 0.13 | 0.18 | 0.14 | 0.10 | 0.24 | 0.34 | 0.30 | 0.30 | 0.13 | 0.25 | 0.27 | 0.29 | 0.71 | 0.87 | 1.00 |  |
| C ch | -0.05 | 0.14 | 0.09 | 0.29 | 0.11 | 0.24 | 0.17 | 0.24 | 0.11 | 0.29 | 0.21 | 0.28 | -0.05 | 0.49 | 0.62 | 1.00 |

| **Pairwise Frequency Table** | | | | | | | | | | | | | | | | |
| --- | --- | --- | --- | --- | --- | --- | --- | --- | --- | --- | --- | --- | --- | --- | --- | --- |
|  | **T T1** | **T T2** | **T T3** | **T ch** | **E T1** | **E T2** | **E T3** | **E ch** | **P T1** | **P T2** | **P T3** | **P ch** | **C T1** | **C T2** | **C T3** | **C ch** |
| T T1 | 152 |  |  |  |  |  |  |  |  |  |  |  |  |  |  |  |
| T T2 | 152 | 152 |  |  |  |  |  |  |  |  |  |  |  |  |  |  |
| T T3 | 152 | 152 | 152 |  |  |  |  |  |  |  |  |  |  |  |  |  |
| T ch | 152 | 152 | 152 | 152 |  |  |  |  |  |  |  |  |  |  |  |  |
| E T1 | 103 | 103 | 103 | 103 | 103 |  |  |  |  |  |  |  |  |  |  |  |
| E T2 | 105 | 105 | 105 | 105 | 102 | 105 |  |  |  |  |  |  |  |  |  |  |
| E T3 | 104 | 104 | 104 | 104 | 102 | 103 | 104 |  |  |  |  |  |  |  |  |  |
| E ch | 103 | 103 | 103 | 103 | 103 | 102 | 102 | 103 |  |  |  |  |  |  |  |  |
| P T1 | 136 | 136 | 136 | 136 | 98 | 101 | 99 | 98 | 144 |  |  |  |  |  |  |  |
| P T2 | 137 | 137 | 137 | 137 | 98 | 101 | 99 | 98 | 144 | 145 |  |  |  |  |  |  |
| P T3 | 134 | 134 | 134 | 134 | 97 | 100 | 98 | 97 | 141 | 142 | 142 |  |  |  |  |  |
| P ch | 136 | 136 | 136 | 136 | 98 | 101 | 99 | 98 | 144 | 144 | 141 | 144 |  |  |  |  |
| C T1 | 130 | 130 | 130 | 130 | 95 | 97 | 96 | 95 | 130 | 131 | 129 | 130 | 137 |  |  |  |
| C T2 | 130 | 130 | 130 | 130 | 95 | 97 | 96 | 95 | 130 | 131 | 129 | 130 | 137 | 137 |  |  |
| C T3 | 130 | 130 | 130 | 130 | 95 | 97 | 96 | 95 | 130 | 131 | 129 | 130 | 137 | 137 | 137 |  |
| C ch | 130 | 130 | 130 | 130 | 95 | 97 | 96 | 95 | 130 | 131 | 129 | 130 | 137 | 137 | 137 | 137 |

| **Matrix of Probabilities** | | | | | | | | | | | | | | | | |
| --- | --- | --- | --- | --- | --- | --- | --- | --- | --- | --- | --- | --- | --- | --- | --- | --- |
|  | **T T1** | **T T2** | **T T3** | **T ch** | **E T1** | **E T2** | **E T3** | **E ch** | **P T1** | **P T2** | **P T3** | **P ch** | **C T1** | **C T2** | **C T3** | **C ch** |
| T T1 | 0.00000 |  |  |  |  |  |  |  |  |  |  |  |  |  |  |  |
| T T2 | 0.00000 | 0.00000 |  |  |  |  |  |  |  |  |  |  |  |  |  |  |
| T T3 | 0.00000 | 0.00000 | 0.00000 |  |  |  |  |  |  |  |  |  |  |  |  |  |
| T ch | 0.99999 | 0.00000 | 0.00000 | 0.00000 |  |  |  |  |  |  |  |  |  |  |  |  |
| E T1 | 0.01189 | 0.03629 | 0.01133 | 0.64549 | 0.00000 |  |  |  |  |  |  |  |  |  |  |  |
| E T2 | 0.06836 | 0.02253 | 0.04142 | 0.21873 | 0.00000 | 0.00000 |  |  |  |  |  |  |  |  |  |  |
| E T3 | 0.25933 | 0.21724 | 0.15814 | 0.45815 | 0.00000 | 0.00000 | 0.00000 |  |  |  |  |  |  |  |  |  |
| E ch | 0.85555 | 0.49350 | 0.79687 | 0.24631 | 0.99999 | 0.00000 | 0.00000 | 0.00000 |  |  |  |  |  |  |  |  |
| P T1 | 0.01534 | 0.08560 | 0.05142 | 0.75104 | 0.00001 | 0.00000 | 0.00026 | 0.12252 | 0.00000 |  |  |  |  |  |  |  |
| P T2 | 0.02246 | 0.00842 | 0.02050 | 0.26592 | 0.00078 | 0.00000 | 0.00006 | 0.00058 | 0.00000 | 0.00000 |  |  |  |  |  |  |
| P T3 | 0.06096 | 0.00557 | 0.02250 | 0.06726 | 0.00449 | 0.00000 | 0.00001 | 0.00006 | 0.00000 | 0.00000 | 0.00000 |  |  |  |  |  |
| P ch | 0.56017 | 0.01868 | 0.13572 | 0.00559 | 0.91679 | 0.00542 | 0.00503 | 0.00001 | 1.00000 | 0.00000 | 0.00000 | 0.00000 |  |  |  |  |
| C T1 | 0.01324 | 0.15688 | 0.52233 | 0.06739 | 0.03798 | 0.00756 | 0.02385 | 0.06592 | 0.11462 | 0.05606 | 0.00409 | 0.02114 | 0.00000 |  |  |  |
| C T2 | 0.07305 | 0.02828 | 0.49792 | 0.97889 | 0.01574 | 0.00010 | 0.00390 | 0.00221 | 0.01401 | 0.00003 | 0.00002 | 0.00003 | 0.00000 | 0.00000 |  |  |
| C T3 | 0.13265 | 0.04313 | 0.10229 | 0.25319 | 0.02035 | 0.00055 | 0.00326 | 0.00345 | 0.13157 | 0.00362 | 0.00212 | 0.00084 | 0.00000 | 0.00000 | 0.00000 |  |
| C ch | 0.60824 | 0.11078 | 0.30958 | 0.00088 | 0.28996 | 0.01806 | 0.08819 | 0.01766 | 0.20334 | 0.00064 | 0.01792 | 0.00137 | 0.57647 | 0.00000 | 0.00000 | 0.00000 |

1. Team members’ (A and B) n Power interaction effect on average punishment in winners (upper panel) and losers (lower panel) in the team condition

Supplement references

Martin, P., Bateson, P., 1993. Measuring behaviour: An introductory guide, 2 ed. Cambridge University Press, New York.
